# Triplet‐Induced Lesion Formation at CpT and TpC Sites in DNA

**DOI:** 10.1002/chem.201903573

**Published:** 2019-10-25

**Authors:** Julia Gontcharov, Lizhe Liu, Bert M. Pilles, Thomas Carell, Wolfgang J. Schreier, Wolfgang Zinth

**Affiliations:** ^1^ Lehrstuhl für BioMolekulare Optik Fakultät für Physik and Center for Integrated Protein Science Munich CIPSM Ludwig-Maximilians-Universität München Oettingenstr. 67 80538 München Germany; ^2^ Center for Integrated Protein Science am Department Chemistry Ludwig-Maximilians-Universität München Butenandtstraße 5–13 81377 München Germany

**Keywords:** cyclobutane pyrimidine dimer, dna damage, photosensitization, time-resolved ir spectroscopy, uv irradiation

## Abstract

UV irradiation induces DNA lesions particularly at dipyrimidine sites. Using time‐resolved UV pump (250 nm) and mid‐IR probe spectroscopy the triplet pathway of cyclobutane pyrimidine dimer (CPD) formation within TpC and CpT sequences was studied. The triplet state is initially localized at the thymine base but decays with 30 ns under formation of a biradical state extending over both bases of the dipyrimidine. Subsequently this state either decays back to the electronic ground state on the 100 ns time scale or forms a cyclobutane pyrimidine dimer lesion (CPD). Stationary IR spectroscopy and triplet sensitization via 2′‐methoxyacetophenone (2‐M) in the UVA range shows that the lesions are formed with an efficiency of approximately 1.5 %. Deamination converts the cytosine moiety of the CPD lesions on the time scale of 10 hours into uracil which gives CPD(UpT) and CPD(TpU) lesions in which the coding potential of the initial cytosine base is vanished.

## Graphical abstract

**Triplet–triplet energy transfer** (TTET) from 2′‐methoxyacetophenone (2‐M) after UVA irradiation induces cyclobutane pyrimidine lesions (CPDs) at dipyrimidine sequences. CPD formation occurs via a localized thymine triplet state and a biradical intermediate. Deamination on a time scale of 10 h converts the deoxy‐cytosine moiety of the CPDs into deoxyuridine.

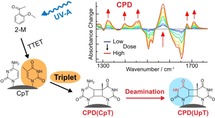

## Introduction

Exposure to sunlight is a constant threat for the integrity of the genetic code in living cells. The damaging effects can be ascribed to the ultraviolet part of the solar spectrum inducing the direct or indirect formation of different types of DNA lesions. The most abundant lesions occur between neighboring pyrimidine bases (thymine and cytosine) and include the formation of cyclobutane pyrimidine dimers (CPDs) and pyrimidine (6‐4) pyrimidone photoproducts.^1^ These lesions are known to lead to mutations in the genome and are regarded as precursors of skin cancer.^2^ The main reason for this lesion formation process is that DNA absorbs UV radiation, which leads to the photoexcitation of the pyrimidine bases. The so‐formed excited state bases have multiple reaction and deactivation channels. Research over the last years was able to uncover most of the photophysical and photochemical processes that occur after photoexcitation of thymine bases (T) in all thymine sequences. These involve excited state deactivation processes including charge‐transfer events and the above‐mentioned formation of DNA lesions.^3^, ^4^, ^5^ In particular it was found that under UVC irradiation direct excitation of thymine bases in TT sequences is the major pathway forming CPD lesions within 1 ps via a singlet excited state.^6^, ^7^ Population of the triplet state via intersystem crossing can also yield CPD lesions but is limited by the overall triplet yield on the order of 0.01 and a comparatively small quantum yield for dimerization to occur from this state.^8^, ^9^

While the UV‐induced formation of DNA lesions at TT sequences is now well established, very little is known about the mechanisms leading to the much more mutagenic lesion formation processes that occur at cytosine (C) containing dipyrimidine sites. As in TT sequences, lesion formation in TC and CT sequences can either occur via direct absorption of UV light or alternatively via indirect processes after absorption of light by other absorbers. The latter is most important in the UVA range were the direct absorption of individual DNA bases is strongly reduced.^5^ Instead, cofactors or biosynthetic intermediates to DNA can function as absorbers for the incoming radiation. If these chromophores are adjacent to the genetic material, they can induce triplet states in DNA via triplet–triplet energy transfer (TTET).^10^, ^11^ Indeed, it is known that TTET leads to the formation of CPDs but not to (6‐4) photoproducts in small model systems as well as in DNA.^1^ The wide variety of compounds that can act as photosensitizers includes aromatic ketones and fluoroquinolones.^12^, ^13^ Another class of potential threats are internal photosensitizers within DNA itself. These include oxidatively generated thymine derivatives as 5‐formyluracil as well as the pyrimidone sub‐unit of the (6‐4) photoproduct.^14^, ^15^ The latter was titled a trojan horse, leading to the possible accumulation of triplet‐induced DNA lesions.^16^, ^17^ Recently, CPD lesion formation was also observed via chemiexcitation. In the latter, UV exposure within melanocytes leads to the formation of oxidation products of melanin, forming activated carbonyls in the triplet excited state. They may induce CPDs long after UV exposure via TTET.^18^

In contrast to irradiation with wavelengths below 300 nm, which are shielded at the present time by the ozone layer, the high transparency of the atmosphere in the UVB and UVA range (above 310 nm) leads to strong irradiation intensities in this spectral range at the surface of the earth and consequently to widespread sensitizing induced DNA damage. Because of the higher radiation intensity in the UVA range compared to the UVB range UVA‐induced CPDs represent a few percent of the overall CPD load in skin tissue.^1^

Direct and indirect UV‐induced lesion formation reactions at thymine bases are favored by the fact that T has the energetically lowest triplet state.^19^ In a more recent study a value of about 270 kJ mol^−1^ was derived as the functional triplet energy of thymine in DNA.^20^ Therefore, any chemical with a higher triplet state energy has to be considered as a potential triplet sensitizer. Efficient energy transfer processes either form outside lying chromophores or even within the DNA duplex lead to an excitation energy transfer to T and consequently to the predominant population of its excited states. This provides DNA lesions at TpT, CpT and TpC sites. Particularly mutagenic are, however, the lesions that are formed at C‐containing sites.^21^ This is due to deamination reactions which convert the C derived part of the UV lesions into the uracil (U) derived products such as CPD(TpU) and CPD(UpT).^22^, ^23^ The U‐containing lesions are subsequently replicated into TpT sites.^24^, ^25^ This C to T conversion is, despite the presence of fast and dedicated repair systems, in fact the major reason for the high mutagenicity of UV and visible light.

To our knowledge the formation of the CPD lesion in the mixed di‐pyrimidines CpT and TpC has never been studied in detail by time‐resolved techniques. While femtosecond and nanosecond‐IR techniques were used to study the singlet and the triplet reaction channel of TpT or (dT)_18_,^3^, ^6^, ^7^, ^8^ these techniques have not been used for the investigation of the reaction dynamics of CpT and TpC. Additionally, the photophysics of individual thymine and cytosine derivatives are still the topic of current research (e.g., see refs.^26^, ^27^, ^28^, ^29^, ^30^), building the foundation for a detailed understanding of photoreactions in the corresponding dipyrimidines.

Interestingly, it was found for duplex DNA that the proportion of TT CPDs induced via TTET is unambiguously higher than the simple statistical prediction assuming an equal reactivity of each thymine involved in TT, CT or TC steps.^10^ As potential explanation for this finding it was suggested that the nature of the adjacent base modifies the triplet energy value of thymine, probably as a result of stacking. Thymine in TT might then be a better target relative to thymine in TC and CT sequences. Additionally delocalization of the involved excited states could be sequence dependent and contribute to different yields.

The formation of CPD lesions via the triplet channel has mainly been the subject of theoretical studies for isolated dimers (e.g., refs. 31, 32). A more recent theoretical study on the sequence dependence of photodimerization pathways in TpdC and dCpT dinucleotides focused on the direct formation of CPD and (6‐4) lesions via charge transfer and exciton states.^33^

In this paper we use time‐resolved and stationary IR spectroscopy to unravel the mechanisms that lead to lesion formation at C‐containing dipyrimidine sites from the thymine triplet state. Time‐resolved IR spectroscopy resolves the evolution of the triplet state via a biradical intermediate. Stationary IR studies allow the characterization of lesion formation after triplet photosensitization in the UVA range and the subsequent deamination reaction of the C‐containing CPD lesions. With this combined approach we cover a time range from nanoseconds to hours and are able to unravel not only the initial mechanisms that lead to lesion formation at C‐containing dipyrimidine sites but also the follow‐up processes that are responsible for the high mutagenic potential of these particular DNA sequences.

## Results and Discussion

**Figure 1:**
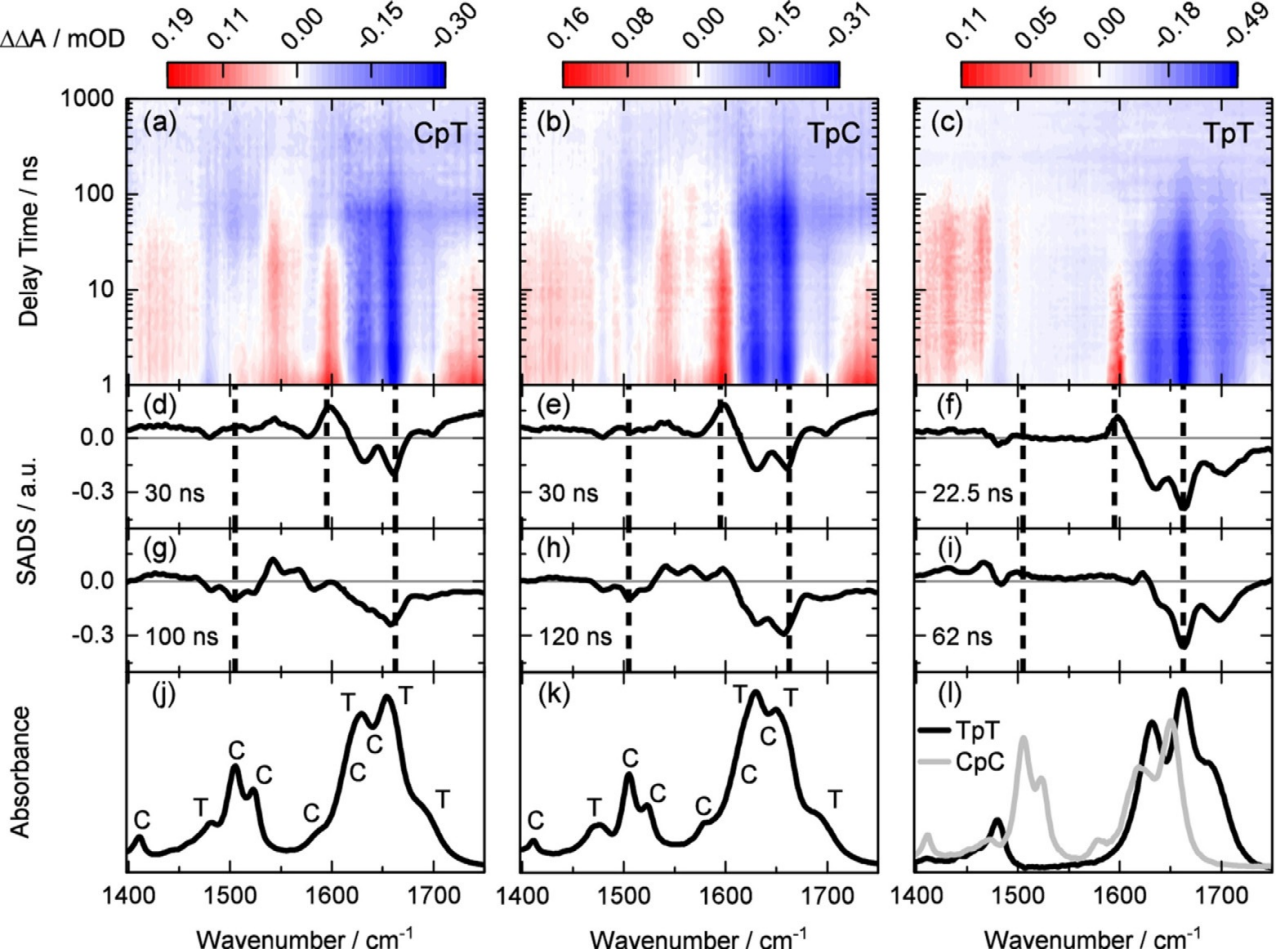
Time‐resolved absorption spectra of the dinucleoside monophosphates CpT (first column), TpC (second column) and TpT (third column, data from ref. 9). Upper part: Time‐resolved IR absorption changes induced by excitation at 250 nm. Second and third row: Species associated difference spectra (SADS) obtained from a global fitting of the data using a sum of two exponentials and a residual difference spectrum. Spectral positions characteristic for intermediate states are marked by dashed vertical lines. Bottom: Ground state absorption spectra of the different dinucleoside monophosphates. Band positions of specific vibrational modes of thymine (T) and cytosine (C) are marked.

In Figure 1 we present the IR absorption dynamics of different dinucleoside monophosphates (CpT, TpC and TpT at concentrations of 5 mm) induced by excitation pulses at 250 nm (pulse duration 3 ns) on the nanosecond time scale. At this wavelength both pyrimidines, thymine (T) and cytosine (C), absorb photons via a low‐lying π–π* transition. Directly after the absorption a number of ultrafast processes occurs on the picosecond time scale, such as internal conversion (IC), direct CPD formation,^6^ cooling of the vibrationally hot bases,^4^ formation and decay of transient radical pair states.^3^ These early dynamics are not visible in the investigated nanosecond time range. In addition, there is the formation of a small amount of localized thymine triplet states via intersystem crossing (ISC).^6^, ^8^, ^34^ The evolution of the triplet states and subsequent reactions on the nanosecond time scale are displayed in Figure 1. In the upper panels the absorption change ΔΔA is plotted in a color code as a function of the delay time and probing wavenumber for CpT (a), TpC (b) and TpT (c). For all samples the absorption change at very late delay times in the microsecond range has been subtracted from the data in order to remove the effects from the heating of the surrounding water, from product formation (discussed below) and to highlight the changes on the nanosecond time scale.

The absorption changes observed on the nanosecond time scale exhibit similar features for all three samples. (i) The first 10 ns are dominated by an absorption decrease (blue) at the position of the C=O stretching modes of thymine between 1610 cm^−1^ and 1720 cm^−1^, a range of strong initial absorption of the parent molecules. Additionally, a pronounced absorption increase (red) is found around 1600 cm^−1^ where the localized triplet state ^3^T of thymine has its characteristic absorption band.^8^, ^35^ The triplet bands disappear within ca. 50 ns. (ii) For all samples further absorption changes are observed on the 100 ns time scale. In this time period CpT and TpC show pronounced absorption changes between 1500 cm^−1^ and 1600 cm^−1^, which are absent in TpT. Towards even later delay times the absorption changes stay essentially constant. More details on the transient absorption changes become evident from a global analysis of the data using a sum of two exponential functions (time constants *τ_i_*, *i*=1 and 2) together with a residual difference spectrum. The corresponding time constants are *τ_1_*≈30 ns and *τ_2_*≈100 ns for CpT, *τ_1_*≈30 ns and *τ_2_*≈120 ns for TpC and *τ_1_*=22.5 ns and *τ_2_*=62 ns for TpT (ref. 9). The fit amplitudes and the decay associated difference spectra (DADS) for CpT and TpC are given in Figure S1 in the Supporting Information.

**Figure 2:**
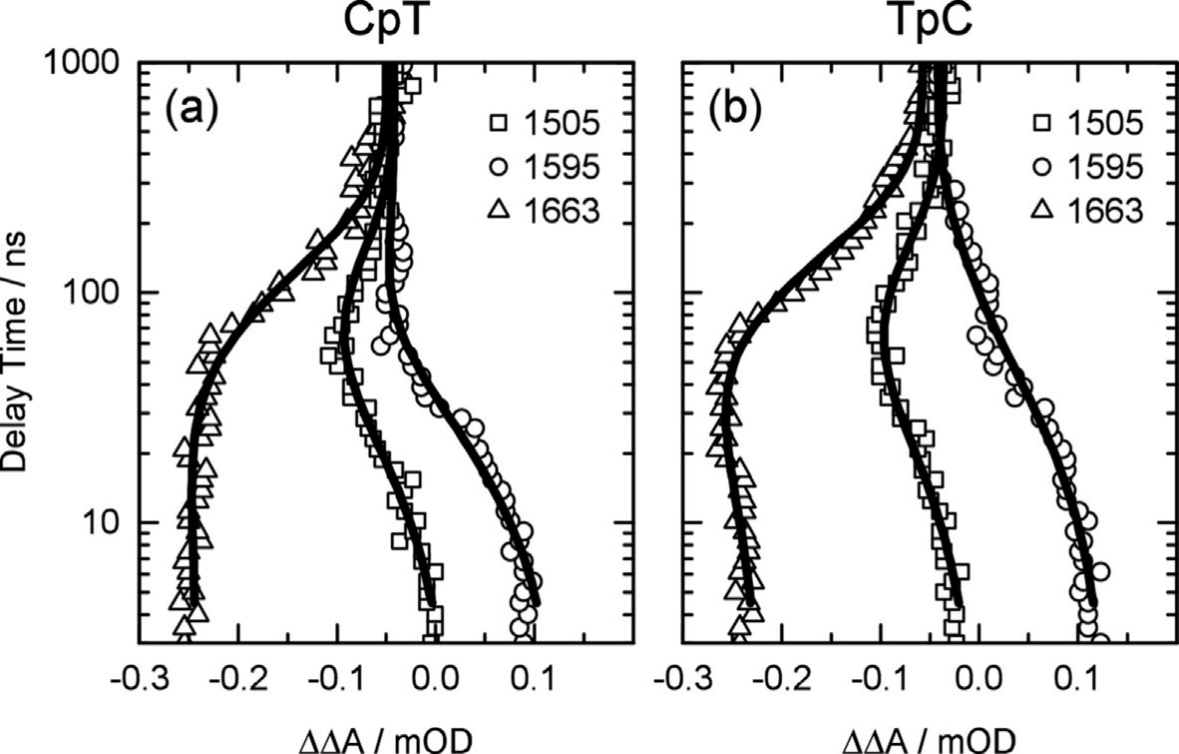
Absorbance changes ΔΔA recorded at the indicated probing wavenumbers (symbols) as a function of the delay time together with the fit curves (solid). The wavenumbers were chosen to show the characteristic evolution of different intermediate states. At 1505 cm^−1^ the bleach and recovery of the cytosine absorption is seen (squares). At 1595 cm^−1^ the decay of the triplet state ^3^T (circles) is observed. The final recovery of the ground state absorption due to the decay of the biradical state is seen at 1663 cm^−1^ (triangles).

Recently published results on the all‐thymine oligomer (dT)_18_, the dinucleoside monophosphate TpT and the thymidine monophosphate TMP allow to obtain a molecular interpretation of the reaction dynamics of CpT and TpC.^3^, ^9^, ^29^ The triplet lifetimes of thymine and the monomer TMP depend on the quenching ability of the surrounding (including oxygen and self‐quenching reactions) with time constants extending to the microsecond range for diluted, nitrogen purged solutions.^35^, ^36^, ^37^ The triplet quenching rate constant for TMP in aqueous solution by molecular oxygen is expected to result in a lifetime of 500 ns.^8^ In (dT)_18_ and TpT the lifetimes of localized triplet states are significantly reduced to about 10 ns and 22.5 ns, respectively, due to efficient quenching of the triplet state ^3^T by adjacent thymine bases. The quenching reaction includes the formation of a biradical intermediate state, which predominantly decays to the electronic ground state with τ_2_=62 ns.^8^, ^9^ The corresponding species associated difference spectra (SADS) for TpT (Figure 1 f, i) show the presence of the absorption band at 1600 cm^−1^ characteristic for ^3^T,^35^ and the bleach of the original thymine bands. The second intermediate, the biradical BR(TpT), shows an SADS (Figure 1 i) with a positive band at 1623 cm^−1^. The SADS of the initial intermediate (Figure 1 d,e) of both C‐containing dinucleoside monophosphates CpT and TpC are very similar to the SADS assigned to the triplet state ^3^T of TpT. There is no absorption decrease in the spectral range assigned originally to bands of cytosine (i.e., around 1510 cm^−1^). Therefore, cytosine is not involved in this first intermediate state of CpT and TpC. This observation suggests that the first intermediate state observed in the two C‐containing dinucleoside monophosphates is the triplet state ^3^T localized on thymine. The decay of this triplet state occurs with ca. 30 ns for both CpT and TpC (see Figure 2 a and b), suggesting that cytosine is able to quench ^3^T in both samples.

The SADS of the second intermediate state formed upon the decay of ^3^T in CpT and TpC show an absorption decrease at positions where cytosine absorbed originally. For instance, the two cytosine bands at 1505 cm^−1^ and 1522 cm^−1^ (see Figure 1 j and k) are well seen as bleaches in the SADS (Figure 1 g and h). At the same time absorption bands appear around 1550 cm^−1^ and the bleaches of the thymine bands persist. These features indicate that the second intermediate state involves both bases of the dinucleoside monophosphates, thymine as well as cytosine. Thus, cytosine adjacent to a thymine not only quenches ^3^T but takes part in a molecular construct involving both constituents. Based on these findings we propose that for CpT and TpC a biradical‐like state exists, similar to the one recently postulated for (dT)_18_ and TpT.^8^, ^9^ This assignment is in agreement with quantum chemical calculations on the IR absorption spectra of biradical structures of BR(CpT) and BR(TpC). Calculations for CpT are given in Figure S2 in the Supporting Information (please note the systematic frequency shifts between measured and calculated frequencies). Instead of two ring vibrations of cytosine around 1527 cm^−1^ and 1550 cm^−1^ present in the calculation in the ground state (panel a) and triplet state (panel b) one observes in the biradical state (panel c) a vibrational mode around 1545 cm^−1^ that is blue shifted compared to the 1527 cm^−1^ band and exhibits a higher intensity. The later findings explain the transient disappearance of the original C bands and the increased absorption in the 1550 cm^−1^ range as observed in the time‐resolved experiment in Figure 1. The decays of the biradical states of CpT and TpC are somewhat slower than that of TpT. Instead of *τ_2_*=62 ns for TpT we find *τ_2_*≈100 ns and 120 ns for CpT and TpC, respectively. The evolution of these intermediates is well seen in Figure 2 a, b, where the time dependences of the absorption changes ΔΔA are plotted for selected wavenumbers. The absorption traces recorded at 1595 cm^−1^ represent the decay of ^3^T and at 1505 cm^−1^ the bleach and recovery of the cytosine absorption. The decay of the biradical and the final recovery of the ground state absorption can be seen best at 1663 cm^−1^. The reaction model of the different dinucleoside monophosphates is given in Scheme 2 (left part) by the example of CpT.

The time‐resolved experiments clearly show that most of the bases in the triplet state ^3^T relax to the initial ground state. For the given precision of the time‐resolved experiments we are not able to observe directly the formation of CPD lesions via the triplet state. This is not surprising considering the low triplet yield after excitation at 250 nm and the small efficiency for CPD formation via the triplet channel (4 % for TpT,^9^). In order to gain information on the product formation we performed stationary experiments with extended illumination and with selective excitation of the thymine triplet by triplet‐triplet energy transfer (TTET) from a sensitizing molecule.

**Scheme 1:**
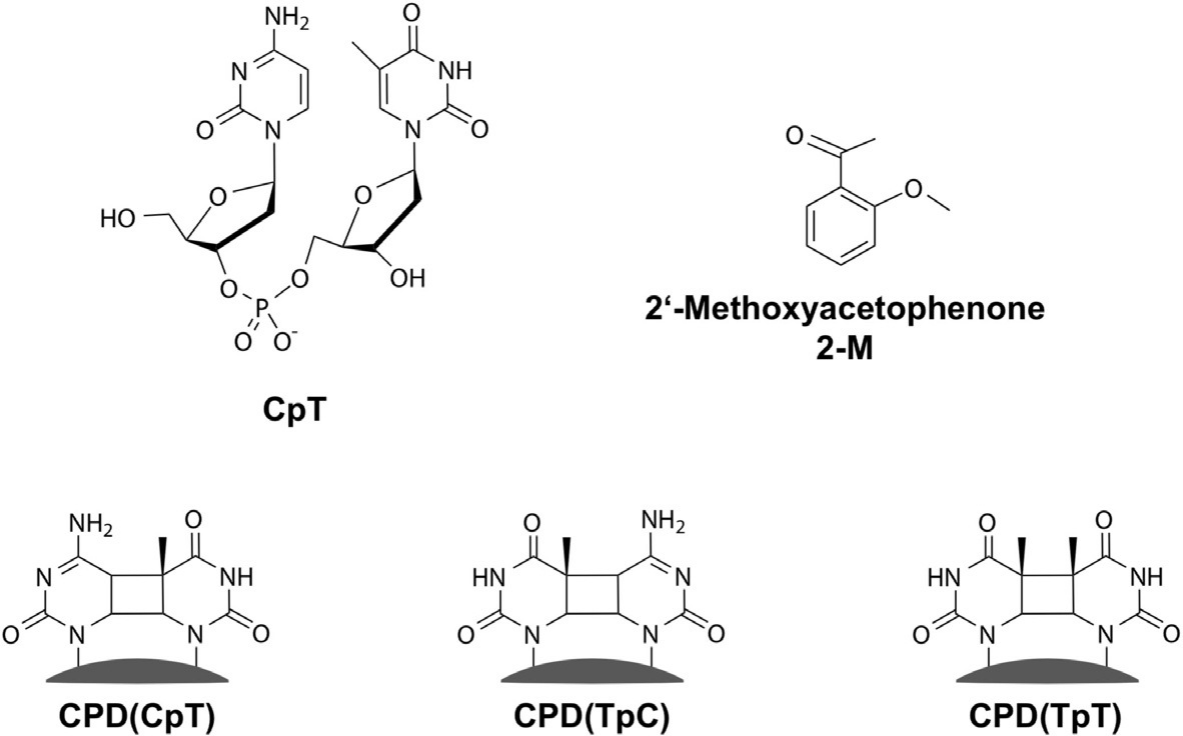
Molecular structures of the dinucleoside monophosphate CpT, the sensitizing molecule 2′‐methoxyacetophenone (2‐M) (upper part) and the CPD lesions of the different dipyrimidines studied.

**Scheme 2:**
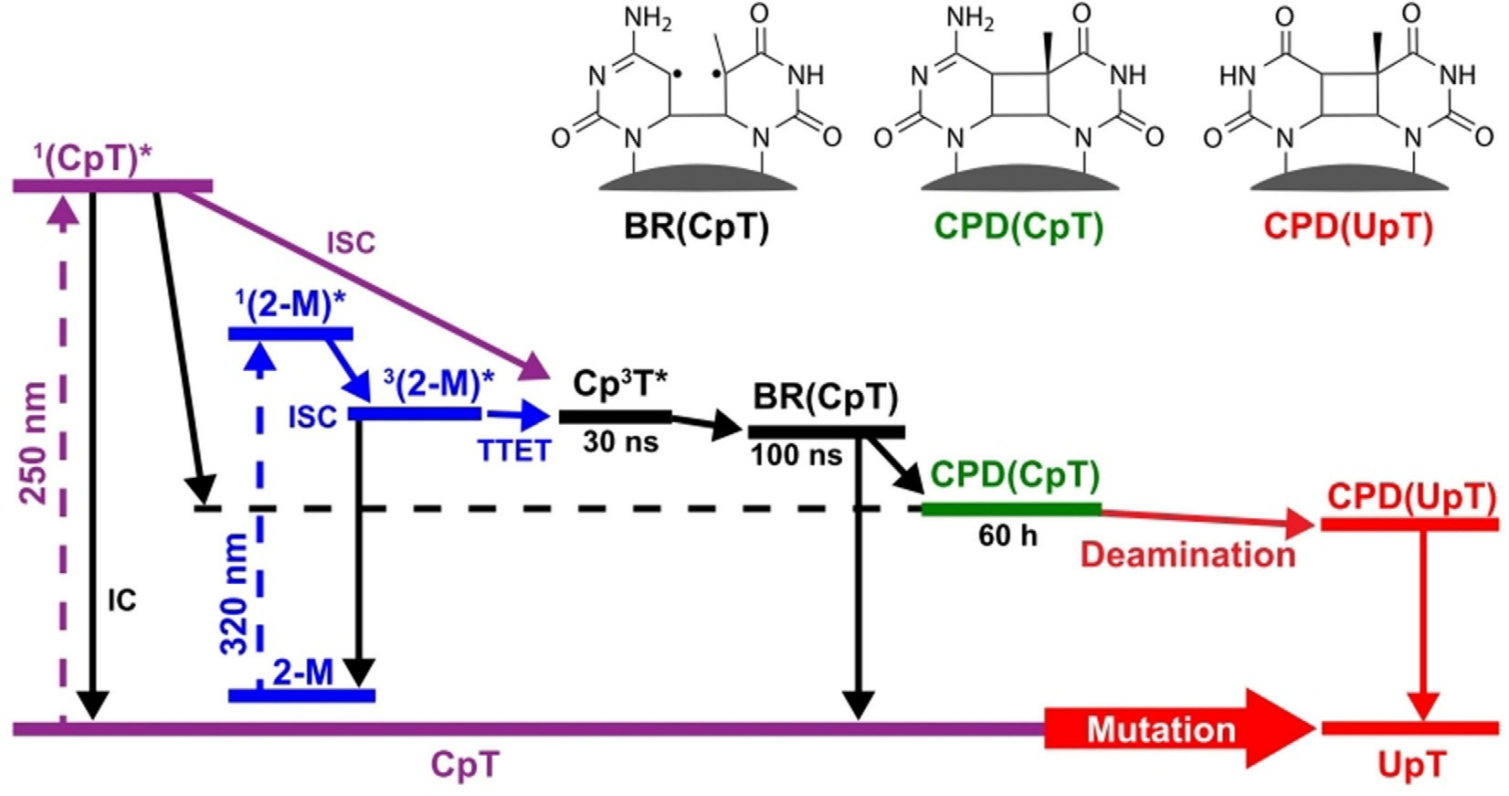
Reaction model for the dinucleoside monophosphate CpT. The left part of the reaction model shows two possible excitation modes of the triplet state Cp^3^T. After the formation of the triplet state the further reaction evolves via the biradical state BR(CpT). While the predominant part of the excited molecules decays to the ground state, a small fraction proceeds to the CPD of CpT and via subsequent deamination to the CPD form of UpT.

Recently, it has been shown that the triplet sensitizer 2′‐methoxyacetophenone (2‐M, see Scheme 1) is an excellent tool to excite the triplet state ^3^T of thymine in DNA oligomers.^9^, ^38^ 2‐M has a large absorption cross‐section in the UVA, a good intersystem crossing (ISC) and a very efficient TTET to thymine (see blue part in the reaction model of Scheme 2). With two experiments (see Supporting Information, Figure S3 and S4), we address the question whether the employed triplet sensitizer 2‐M may excite the triplet state of cytosine in a dinucleoside monophosphate by TTET or may induce photo‐destruction of cytosine. Time‐resolved IR measurements on a solution of 2‐M and CpC (Figure S3) show that cytosine in a dimeric form is not able to quench the triplet state of 2‐M. Thus, there is no reactive excitation of the cytosine triplet via TTET from 2‐M. The illumination at 320 nm of a mixture of 2‐M and CpC (for a comparison to the other dinucleoside monophosphates see Figure S4) did not lead to a detectable absorption bleach in the range of the cytosine bands (around 1520 cm^−1^) which would be an indication for the destruction of cytosine. The lack of both ^3^C triplet generation and decomposition of cytosine in stationary experiments together with the time‐resolved experiments shown in Figure 1 allow us to conclude that the reactions observed in CpT and TpC after TTET from 2‐M are induced by the thymine triplet ^3^T.

**Figure 3:**
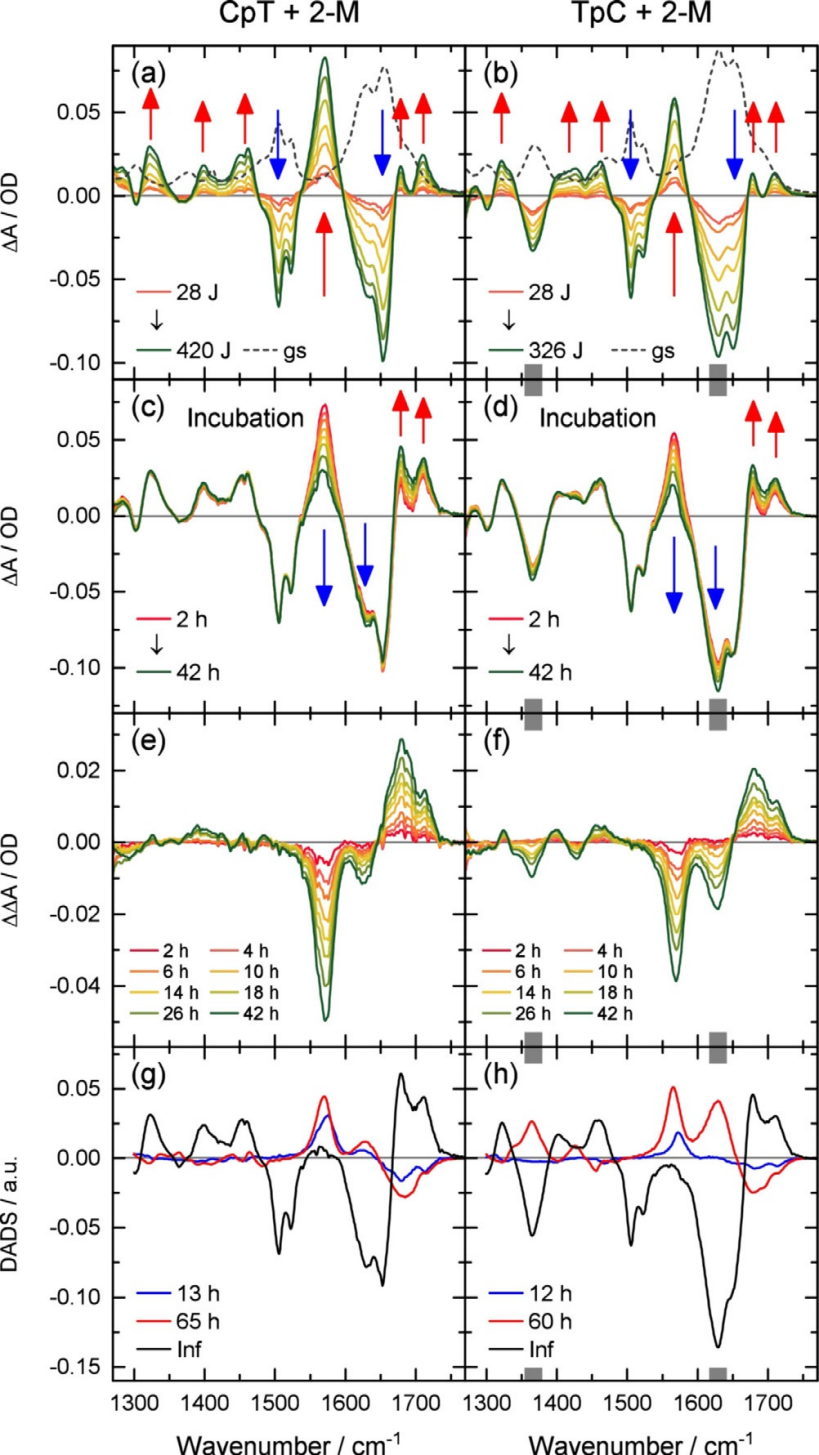
FTIR spectra of CpT (first column) and TpC (second column) upon long‐term illumination at 320 nm exciting the dinucleoside monophosphates via the triplet state of 2‐M. Extended illumination (difference spectra in a, b) leads to the disappearance of C and T absorption bands and the formation of new bands, which can be assigned to the CPD lesion. The dashed curves show the scaled absorption spectra of CpT and TpC. c to f: After the illumination period further spectral changes on the time scale of many hours are observed (c and d, double difference spectra e and f). The analysis of the absorption changes with two exponential functions shows the decay associated difference spectra (DADS) and the corresponding time constants given in panels g and h. The features around 1370 cm^−1^ and 1640 cm^−1^ (gray bars) may be influenced by concentration dependences.

In Figure 3 we present the IR absorption changes obtained upon high‐dose irradiation at 320 nm of solutions containing the sensitizer 2‐M (5 mm) and either the dinucleoside monophosphate CpT (10 mm, a) or TpC (10 mm, b) using irradiation doses up to the 400 J range. In both samples one observes the build‐up of IR absorption changes indicative for the formation of a reaction product (see Figure 3 a, b). (i) There is the decay of the original ground state absorption bands of cytosine and thymine (1475 cm^−1^ to 1530 cm^−1^ and 1600 cm^−1^ to 1680 cm^−1^). (ii One observes absorption increases in the range of the C=O stretching modes at 1678 cm^−1^, 1710 cm^−1^ and between 1250 cm^−1^ and 1470 cm^−1^. The latter show characteristic signatures of the CPD lesion as found in TpT.^6^ (iii) Most prominent is the strong band appearing at 1570 cm^−1^. The appearance of this band also points to the formation of the CPD since quantum chemistry calculations allow to assign this band to a C4=N3 vibrational mode of cytosine in a CPD lesion (see Figure S2).

**Figure 4:**
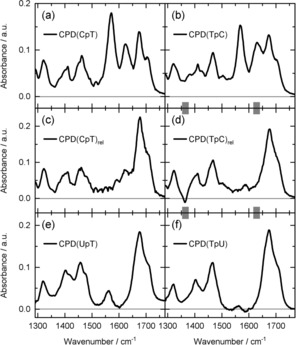
IR spectra assigned to the different CPD states generated via the triplet sensitizer 2‐M. The CPD spectra for CpT (a) and TpC (b) are calculated using the illumination induced absorption changes of Figure 3 a and b. At the end of the incubation period one obtains the absorption spectra shown in panels c and d. Panels e and f: CPD spectra of corresponding uracil‐containing dinucleoside monophosphates are in good agreement with the spectra at the end of the incubation period (c and d). Around 1370 cm^−1^ and 1640 cm^−1^ (gray bars) the spectra may be influenced by concentration dependences.

Apparently the CPD lesions (CPD(CpT) and CPD(TpC), for structures see Scheme 1, lower part) are formed in a reaction sequence (see Scheme 2) via the triplet state ^3^T of thymine. Whether CPD formation occurs directly from the localized triplet ^3^T or from the biradical (e.g. BR(CpT)) cannot be deduced from the stationary experiments. However, it is very likely that CPD formation originates from the biradical state where the excitation extends over both nucleosides and where one bond of the cyclobutane ring is already formed (see Scheme 2).^8^ The CPD spectra CPD(CpT) and CPD(TpC) of the two compounds are given in Figure 4 a, b.

For TpT it has been shown in the literature that the formation yield of the CPD lesion CPD(TpT) from the triplet state ^3^T of thymine occurs with an efficiency of about 4 %.^9^ Using a similar procedure (modifications see the Supporting Information) we obtain a rough estimation of the yield for the formation of the CPD lesion via the triplet channel on the order of 1.5 % ±0.5 %.

The conversion of a major fraction of CpT and TpC to the corresponding CPD lesions (see Figure 3 a and b) required an absorbed energy in the range of 400 J for the investigated volumes of 1.3 mL at the given concentrations (10 mm). After the illumination period, IR absorption measurements have been performed on the time scale of many hours (incubation). These measurements reveal an additional evolution of the IR spectra. The corresponding absorption difference spectra are shown in Figure 3 c and d. The strong band around 1570 cm^−1^ is assigned to cytosine‐containing CPD lesions and decreases for both CpT and TpC on a time scale of several tens of hours. Simultaneously, there is a decay of the absorption around 1620 cm^−1^ and a pronounced rise between 1680 cm^−1^ and 1720 cm^−1^. These changes are clearly seen in the panels e and f of Figure 3, where double difference spectra are plotted to illustrate the absorption changes ΔΔA(*t*)=ΔA(*t*)−ΔA(0) after the irradiation period (*t*=0). These double difference spectra together with the absorption spectra of the starting material allow us to estimate the spectra of the final compounds (abbreviated by CPD(CpT)_rel_ and CPD(TpC)_rel,_ see Figure 4 c, d). The time dependence is reproduced by a fit with two exponential functions resulting in time constants of 13 h and 65 h (CpT) and 12 h and 60 h (TpC).

It has been shown in the literature that cytosine may be converted in aqueous surroundings to uracil (U) via deamination.^39^, ^40^, ^41^, ^42^, ^43^ At room temperature this reaction is rather slow. However, deamination is strongly accelerated when the aromatic C5=C6 bond loses its aromatic character. When the C5=C6 bond becomes part of the cyclobutane ring in a CPD lesion the deamination reaction occurs on the time scale of 10 h.^42^, ^44^ This deamination converts the CPD of CpT or TpC to the CPD of UpT and TpU, respectively.^25^, ^45^, ^46^, ^47^, ^48^, ^49^, ^50^ A comparison of the spectra of CPD(CpT)_rel_ and CPD(TpC)_rel_ (see Figure 4 c, d) with the CPD spectra of the corresponding uracil compounds (see Figure 4 e, f) supports this interpretation. Qualitatively, the estimated spectra of CPD(CpT)_rel_ and CPD(TpC)_rel_ agree well with the spectra of CPD(UpT) and CPD(TpU).

The time dependent absorption changes assigned to the deamination reaction can be described by a biexponential decay for both dinucleoside monophosphates with one time constant around 12 h and the other around 60 h. The corresponding DADS of the slow and the fast components shown in Figure 3 g, h (red and blue traces) have similar band positions but differ considerably in the band amplitudes. Different time constants have been found in the literature for the deamination reaction and assigned to the isomeric forms of the dinucleoside monophosphates. The orientational restrictions by the sugar‐phophodiester backbone strongly favors *cis‐syn* arrangements of CPD lesions and prevents the formation of *anti* forms in DNA strands. For single stranded DNA and oligonucleotides *trans‐syn* diastereomers have been observed in their *syn*‐anti and anti‐*syn* conformation.^10^, ^50^, ^51^, ^52^ The proportions of *cis‐syn* vs. *trans‐syn* isomers obtained after photosensitization of dCpT, TpdC and TpT range from 3:1, 1:1 to 7:1, respectively.^11^ The *cis‐syn* form reacted faster, the *trans‐syn* somewhat slower.^44^, ^45^ Known rate constants for the deamination reaction at room temperature and in buffered solution range from 1.7×10^−3^ min^−1^ to 1.4×10^−3^ min^−1^ for the *cis‐syn* isomers and from 1.1×10^−4^ min^−1^ to 3.9×10^−4^ min^−1^ for the *trans‐syn* isomers of dCpdT(CPD) and dTpdC(CPD).^44^, ^45^ The two time constants of about 12 h and 60 h obtained monitoring the IR absorption changes translate into rate constants of 1.4×10^−3^ min^−1^ and 2.8×10^−4^ min^−1^, respectively The latter are well within the range of the above mentioned results, supporting the assignment.

The presented DADS indicate that the isomeric structures show differences in the band positions and intensities. Thereby the amplitudes of the DADS suggest that the *trans‐syn* component is present in a higher yield, seemingly contradicting the earlier results. Yet, while the differences in the band amplitudes may be an indication for the relative compositions, the determination of the absolute amount of the respective isomeric forms cannot be directly deduced from the DADS signals. The irradiation period for the indicated dose on the sample volumes took several hours. This leads to a pronounced underrepresentation of the amount of the faster decaying *cis‐syn* isomers. Additionally, the determination of absolute values for the respective extinction coefficients requires purified samples of the respective CPDs which is hampered by the deamination reaction as well and was not aimed for in the present study.

## Conclusions

In the literature, it had been shown for TT sites that CPD formation after excitation with UVC occurs nearly exclusively via the singlet channel.^5^ While this presumably holds true also in the UVB range, it was shown that an excited triplet state ^3^T, generated for example, by sensitization from the abundant UVA radiation, can also lead to CPD formation in TT sequences.^8^, ^9^ In the present investigation we demonstrated, that the triplet channel via ^3^T also acts in the formation of the highly mutagenic CPD lesions at the mixed dipyrimidine sites TC and CT. In detail we targeted here the formation of CPD lesions in the dinucleoside monophosphates CpT and TpC after UV irradiation using a combination of pump probe spectroscopy spanning the nanosecond time scale and longer time frame IR measurements to fully cover the lesion formation processes under inclusion of the cytosine base (C). On the way to C‐containing CPD lesions, we show that either direct excitation of CpT/TpC sequences (with UV light at 250 nm) or transfer of excitation energy onto such sequences via a triplet sensitizer (2‐M under UVA illumination) leads to selective excitation of the T base, which is rapidly excited into the triplet state. Subsequently the triplet state ^3^T of the C‐containing dipyrimidines is quenched within 30 ns by the neighbouring C base, forming a biradical intermediate state (BR) extending over both bases of the pyrimidine. This biradical intermediate is the key starting point for the formation of the CPD lesion. It either decays with ca. 100 ns back to the electronic ground state or forms the CPD lesion.

The triplet sensitization experiments in the UVA range (with excitation at 320 nm) show that the corresponding CPD lesions are formed with an efficiency of ca. 1.5 %, that is, the build‐up of the CPD via the triplet channel in the mixed dipyrimidines occurs with a smaller efficiency than in TpT (ca. 4 %). Considering the relevance of this results for duplex DNA it is interesting to note, that the derived reaction Scheme provides an alternate explanation for the observation of a reduced quantum efficiency for CPD formation observed for at C‐containing dipyrimidine sequences compared to TT steps after photosensitization of duplex DNA.^10^ The latter could at least partly be due to the involvement of a biradical intermediate and the 2–3 times lower quantum yield found for CPD formation in the cytosine‐containing dinucleoside monophosphates compared to TpT. Additionally, our results clearly show that the CPD lesions of C‐containing dipyrimidine sites can indeed be formed via the thymine triplet ^3^T state. The latter is of special interest as triplet excited states are suggested to play a minor role in comparison to the overall yield of CPD lesion formation in the UVB range.^53^ Yet, the mechanisms responsible for UVA‐induced CPDs are still a matter of debate.^1^ The direct absorption of UVA photons by DNA has been related to stacking of bases in the double helix and it has been suggested that the weak absorption of UVA radiation by DNA is due to states with pronounced charge transfer character.^54^ Yet, if and how those states might directly contribute to CPD formation in the UVA range is unclear.^55^ Notwithstanding, the triplet states of thymine act as precursors of C‐containing CPDs which are considered highly mutagenic. While TT CPDs have been found to be the predominant lesion formed under UVB as well as under UVA irradiation,^56^, ^57^ they mainly act as blocks to replication rather than being a highly mutagenic photoproduct.^2^, ^58^ The reason for the high mutagenicity of C‐containing CPD lesions is that the C base loses its aromatic stabilization, if it is embedded in a CPD lesion.^1^ Without aromaticity, however, the C part rapidly deaminates to give U‐containing lesions. These are either repaired or handled by lesion tolerance polymerases which in both cases gives TpT sequences, thereby manifesting a C to T transition mutation. Monitoring the evolution of the C‐containing dipyrimidines via stationary IR spectroscopy we found that the CPD lesions CPD(CpT) and CPD(TpC) evolve by deamination to the U‐containing CPD lesions CPD(UpT) and CPD(TpU) on the time scale of 10 hours. The different species in the reaction sequence (see Scheme 2) could be identified by the evolution of the IR spectra, where the assignment of representative bands was validated by quantum chemical calculations.

Interesting to note is that the C part of the *cis‐syn* configured CPD(CpT) or CPD(TpC) lesions that will be formed in nature, deaminates subsequently with a lifetime of about 12 h under our non‐cellular conditions to give the corresponding U‐containing lesions CPD(UpT) or CPD(TpU). The *trans‐syn* CPD lesions which cannot be formed in double‐stranded DNA due to the geometrical constrains imposed by the double helix structure, have far slower deamination kinetics of about 60 h as expected. While deamination rates have been found to be somewhat slower in double‐stranded DNA than in the investigated dinucleoside monophosphates,^59^, ^60^ deamination plays a major role in the mutagenic properties of cytosine‐containing CPDs.^1^ Because even a fast‐growing embryonic fibroblast needs about 20 h per cell division, the deamination is consequently an unavoidable event in adult skin cells. Deamination will convert every C‐containing CPD lesion into a U‐containing lesion and hence into a mutagenic event. This is the reason why UV‐induced damage in C‐containing dipyrimidine sequences is a formidable challenge for the integrity of the genome.

## Experimental Section

The dinucleoside monophosphates CpT (2′‐deoxycytidylyl‐(3′‐5′)‐thymidine), TpC (thymidylyl‐(3′‐5′)‐2′‐deoxycytidine), TpT (thymidylyl‐(3′‐5′)‐thymidine) and CpC (2′‐deoxycytidylyl‐(3′‐5′)‐2′‐deoxycytidine) were obtained as dried powder from metabion (Germany), UpT (2′‐deoxyuridylyl‐(3′‐5′)‐thymidine) and TpU (thymidylyl‐(3′‐5′)‐2′‐deoxyuridine) from biomers.net (Germany), the photosensitizer 2′‐methoxyacetophenone (2‐M) from Sigma–Aldrich (Germany). All sample molecules were used without further purification. They were prepared in a buffered (50 mm sodium phosphate Na_2_HPO_4_ and 50 mm potassium phosphate KH_2_PO_4_) solution of D_2_O (Merck; Sigma–Aldrich). For the sensitizer experiments a concentration of 2‐M of 5 mm was used, whereas the concentrations of the DNA bases range from 2.5. to 10 mm as detailed in the text. The structures of the dipyrimidine CpT and of the sensitizer 2‐M are shown in Scheme 1, upper part.

The laser setup for the time‐resolved pump‐probe experiments has been described recently.^3^ The system is based on a femtosecond Ti:sapphire laser amplifier system (Tsunami/Spitfire Pro, Spectra Physics) operated with a repetition rate of 1 kHz, with a pulse duration of ≈120 fs and a central wavelength of 800 nm. Tunable mid‐IR (1280 cm^−1^ to 1750 cm^−1^) probe pulses were generated using a combination of a non‐collinear and a collinear optical parametric amplifier and subsequent difference‐frequency mixing in an AgGaS_2_ crystal.^61^ The mid‐IR pulses transmitted through the sample were spectrally dispersed in a grating spectrometer (Chromex 250 IS, Bruker) and recorded with a 64‐channel MCT array detector (IR‐0144, Infrared Systems Development). The polarisations of the pump and the probe pulses were oriented at the magic angle to avoid artefacts from molecular rotations.

A tunable nanosecond laser‐OPO system (EKSPLA NT242) with a repetition rate of 1 kHz (pulse duration 3 ns) provided the narrow‐band (*Δλ*≈0.1 nm) pump pulses synchronized to the IR pulses from the femtosecond laser system.^62^, ^63^ At 250 nm energies of ca. 3.4 μJ (spot diameter 170 μm*x*170 μm), at 320 nm ca. 6 μJ (spot diameter 160 μmx300 μm) were used.

A flow‐through cuvette (PTFE spacer with a thickness of ca. 110 μm between CaF_2_ windows) and a peristaltic pump were used for the experiments to ensure an exchange of the sample volume between two successive pump pulses. Overall sample volumes of 1–2 mL were used. All time‐resolved measurements were performed at room temperature (23 °C) and at ambient oxygen concentrations in purged sample compartments.

The stationary irradiation experiments were also performed with the nanosecond pulses from the EKSPLA system (elliptical spot 3.5 mmx10 mm, repetition rate 1 kHz, pulse energy up to 20 μJ) as illumination source. The IR spectra were recorded in the aforementioned flow‐through cuvette connected to a standard 4 mmx10 mm fused silica cell (Hellma Analytics, Precision Cell Quartz SUPRASIL) where the sample was illuminated by UV light. The FTIR spectra were recorded with a Bruker IFS66v s^−1^ spectrometer in a purged sample compartment at ambient oxygen concentrations and at 27 °C.

For further analysis of the data the following corrections were applied to the absorption spectra. Subtraction of appropriately scaled spectra of the phosphate buffer, of HDO, of the sensitizer 2‐M and of an acetate buffer (to eliminate acetate traces left over from the synthesis of the dipyrimidines). The resulting spectra represent the ground state absorption spectra of each dipyrimidine. In order to obtain the CPD spectra shown in the panels a, b, e, f of Figure 4, scaled difference spectra recorded after a long irradiation time (e.g. see Figure 3 a and b) were added to the corresponding ground state spectra. The spectra of Figure 4 c and d were determined in a similar manner by adding scaled double difference spectra (Figure 3 e and f) to the corresponding CPD spectra of Figure 4 a and b. The scaling factor was adjusted to avoid an absorption band in the 1570 cm^−1^ range and negative absorption values.

## Conflict of interest

The authors declare no conflict of interest.

## Supporting information

As a service to our authors and readers, this journal provides supporting information supplied by the authors. Such materials are peer reviewed and may be re‐organized for online delivery, but are not copy‐edited or typeset. Technical support issues arising from supporting information (other than missing files) should be addressed to the authors.

Supplementary
