# Supplementary material for: Triplet‐Induced Lesion Formation at CpT and TpC Sites in DNA
Source: Chemistry. 2019 Oct 25;25(66):15164–72. doi: 10.1002/chem.201903573 (PMC6899856; doi:10.1002/chem.201903573)
Supplement: Supplementary file 1 — Supplementary [file CHEM-25-15164-s001.pdf]

CHEMISTRY
A European Journal

Supporting Information

Triplet-Induced Lesion Formation at CpT and TpC Sites in DNA

Julia Gontcharov,[a] Lizhe Liu,[a] Bert M. Pilles,[a] Thomas Carell,*[b] Wolfgang J. Schreier,*[a] and Wolfgang Zinth*[a]

chem_201903573_sm_miscellaneous_information.pdf

Triplet Induced Lesion Formation at CpT and TpC Sites in DNA

Julia Gontcharov, Lizhe Liu, Bert M. Pilles, Thomas Carell, Wolfgang J. Schreier and Wolfgang Zinth

Supporting Information

Contents:

1. Time-resolved spectroscopy of CpT and TpC upon excitation at 250 nm: Decay Associated Difference Spectra

2. Calculated vibrational spectra of CpT and the intermediates reached upon TTET

3. Time-resolved and stationary spectroscopy of sensitization on CpC

4. CPD formation yield from the thymine triplet state ^3T

5. UV-Absorption Spectra of CpT, TpC and 2-M

1. Time-resolved spectroscopy of CpT and TpC upon excitation at 250 nm: Decay Associated Difference Spectra

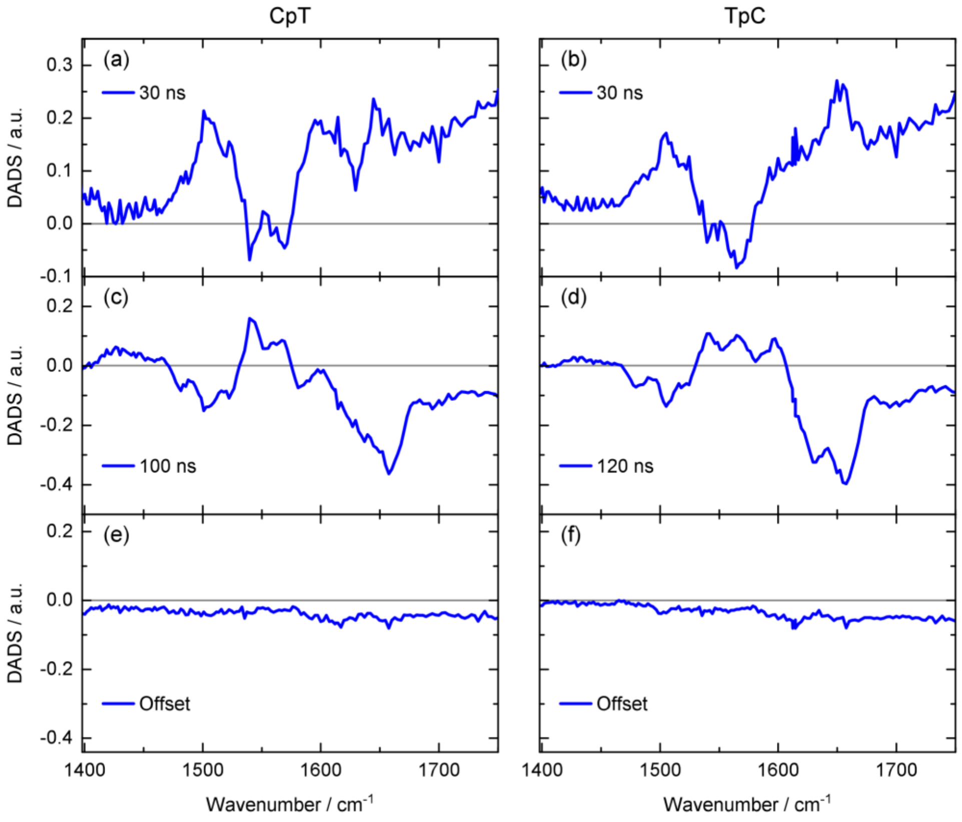

Figure SI1: Decay associated difference spectra (DADS) for CpT and TpC from the analysis of the experimental data presented in Figure 1a and b. The small amplitudes of the final component (Offset) is related with the subtraction of the absorption changes at delay times > 10 µs from the raw data.

2. Calculated vibrational spectra of CpT and the intermediates reached upon TTET

The IR absorption spectra of the dinucleoside monophosphate CpT and its intermediates reached upon TTET were calculated using standard density functional theory (DFT). The calculations were performed with the Gaussian 03 (Revision E.01) quantum-chemical package [1] using the B3LYP density functional [2] with the 6-311G** basis set. Following the geometry optimization computations of harmonic vibrational wavenumbers, normal modes, and IR intensities were carried out using the same functional. Prior to the calculations the labile N3 hydrogen atom and the OH hydrogen atoms were exchanged by deuterium atoms [3]. For a better comparison with the experimental data, the computed stick spectra were convoluted with a Lorentzian function (FWHM of 25 cm^{-1}) [4].

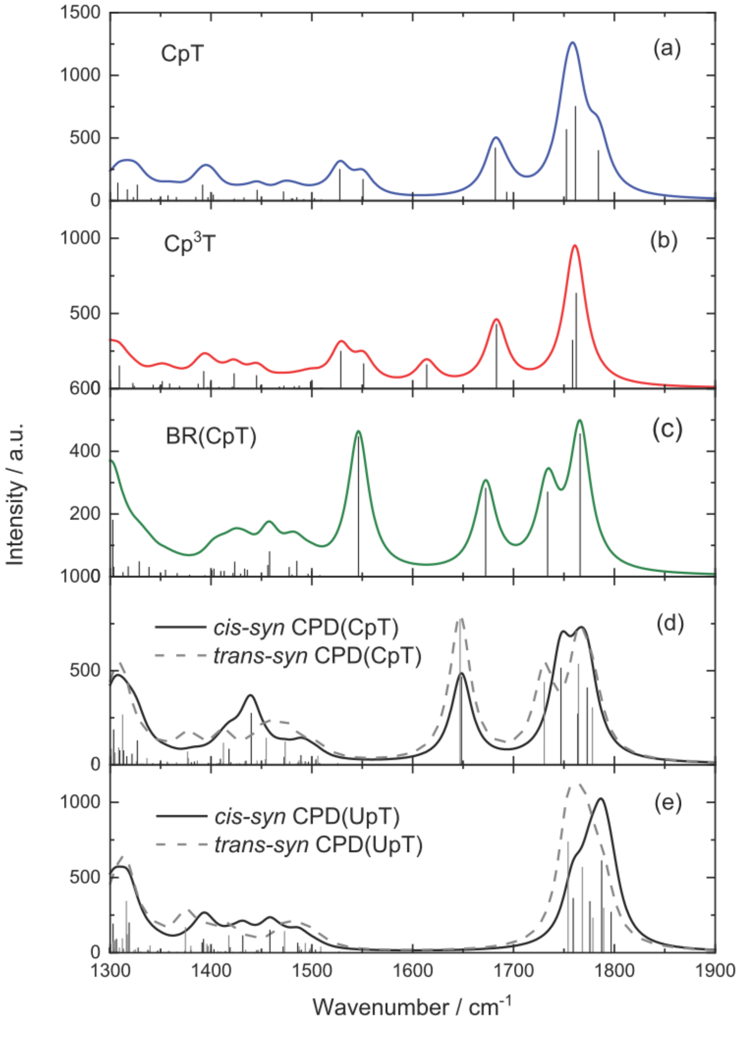

Figure SI2: Calculated vibrational spectra for the original dinucleoside monophosphate CpT (a), the triplet state Cp^3T (b), the biradical state BR(CpT) (c), both *syn*-diastereomers of the CPD(CpT) (d) and the CPD(UpT) (e). The CPD lesion can be formed as *cis-syn* or *trans-syn* isomer based on the relative orientation of the bases. The calculations in panels d and e show the spectra obtained for the respective CPDs in *cis-syn* (anti-anti) conformation and *trans-syn* (syn-anti) conformation.

Cartesian coordiates after geometry optimization

Cartesian Coordinates: CpT

C	0.000000	0.000000	0.000000
N	0.000000	0.000000	1.402587
C	1.181177	0.000000	2.128740
C	2.408296	-0.013639	1.567076
C	2.512518	-0.025355	0.109991
N	1.269021	-0.032715	-0.546790
C	-1.275193	0.136286	2.077521
C	-1.611023	-0.894837	3.158785
C	-2.687695	-0.164916	3.982314
C	-2.442662	1.331842	3.648242
O	-1.299003	1.383605	2.790818
C	-2.245739	2.258955	4.829379
O	-1.291087	1.764157	5.798482
P	0.296486	1.873548	5.712056
O	0.689678	2.247779	7.228869
O	-4.020701	-0.454360	3.564354
C	3.677127	0.014066	2.369537
O	3.546355	-0.028898	-0.530580
O	-1.006800	0.014960	-0.676335
O	0.524050	3.232159	4.912875
C	1.626902	3.428802	3.973592
C	1.082244	4.113450	2.733979
C	1.089635	5.591748	3.140528
O	2.218925	5.735767	4.010690
C	2.601087	4.469893	4.560681
N	1.227909	6.529401	2.032255
C	0.084603	7.333044	1.671034
N	0.262350	8.226498	0.653670
C	1.416896	8.321117	0.029753
C	2.560690	7.516510	0.350723
C	2.409794	6.636917	1.371361
O	-0.961138	7.167648	2.270718
N	1.514277	9.260287	-0.950138
C	4.063948	4.190751	4.249225
O	4.206390	3.967584	2.844916
O	1.023622	0.678614	5.219787
H	3.205839	5.990334	1.720027
H	0.171071	5.878096	3.650415
H	1.781973	3.949689	1.912550
H	0.098421	3.743017	2.451549
H	2.480882	4.504851	5.648472
H	2.094392	2.467714	3.772540
H	5.133007	3.797934	2.651373
H	4.655242	5.055098	4.573424
H	4.385040	3.310087	4.821543
H	-1.936192	3.247844	4.490326
H	-3.182321	2.343879	5.380521
H	-3.329605	1.682056	3.103074
H	-2.569449	-0.356348	5.052307
H	-4.245425	-1.340520	3.864862
H	-2.030589	0.141682	1.290559
H	-1.978442	-1.833676	2.741519
H	-0.733913	-1.093460	3.777607
H	1.298848	-0.039030	-1.558337
H	1.057110	0.042209	3.204350
H	4.352527	-0.785618	2.056065
H	4.208822	0.958418	2.215428
H	3.464591	-0.096125	3.434100
H	1.099167	1.497155	7.674199
H	2.282663	9.247494	-1.597966
H	0.658972	9.715084	-1.227728
H	3.502620	7.615032	-0.170111

Cartesian Coordinates: Cp^{3}T

C	-3.985321	-0.578014	-1.988956
N	-3.589914	-0.415686	-0.609613
C	-4.238065	0.443943	0.218733
C	-5.289566	1.177880	-0.220604
C	-5.676133	0.979868	-1.588387
N	-5.058088	0.156267	-2.407302
C	-2.478079	-1.214586	-0.109774
C	-1.208963	-0.419372	0.216921
C	-0.556016	-1.268588	1.293446
C	-1.754013	-1.905732	2.024483
O	-2.867960	-1.821680	1.129059
O	0.220164	-2.330055	0.659607
P	1.767846	-2.561966	0.976763
O	2.453340	-1.486539	1.730869
C	-2.098506	-1.226031	3.341183
O	-2.185064	0.185467	3.125530
N	-6.755134	1.646921	-2.080358
O	-3.347905	-1.335379	-2.696156
O	2.367310	-2.967953	-0.442303
C	1.909289	-2.494891	-1.731576
C	2.500963	-1.158500	-2.125896
C	4.039974	-1.022693	-1.971467
C	4.199402	0.237970	-1.105902
C	2.870769	0.961277	-1.352485
O	1.910848	-0.108036	-1.352557
N	2.485032	1.967900	-0.382992
C	2.477133	1.676109	0.981867
C	1.674255	2.603381	1.838254
C	1.445857	3.938595	1.389235
N	1.958119	4.210715	0.094389
C	2.291644	3.276701	-0.868617
O	2.429035	3.574420	-2.037092
O	0.869362	4.827818	2.015443
C	1.130175	2.138030	3.132878
O	4.562833	-0.879416	-3.291613
O	1.800912	-3.973701	1.752839
H	-3.868116	0.488646	1.235796
H	-2.300903	-1.972868	-0.871071
H	-1.478980	0.545323	0.650800
H	-0.557944	-0.257767	-0.640295
H	-1.535640	-2.959749	2.224845
H	0.100323	-0.707006	1.954280
H	-2.422120	0.611288	3.954641
H	-3.053535	-1.628086	3.698045
H	-1.318811	-1.462838	4.077480
H	0.820168	-2.449027	-1.734266
H	2.238375	-3.255021	-2.440280
H	2.275944	-1.003523	-3.189857
H	4.468206	-1.901210	-1.481044
H	5.523566	-0.893733	-3.237615
H	2.872243	1.456789	-2.323581
H	5.062617	0.836908	-1.400927
H	4.287696	-0.029696	-0.051793
H	1.766216	5.134530	-0.268358
H	2.567234	0.632939	1.250473
H	0.741942	2.981747	3.702922
H	0.313344	1.415906	2.986644
H	1.903431	1.615267	3.709244
H	2.437768	-3.948169	2.476037
H	-5.815943	1.854478	0.437714
H	-7.139260	2.433745	-1.586764
H	-6.917407	1.576129	-3.072408

Cartesian Coordinates: BR(CpT)

C	-0.671679	3.108693	0.858852
C	-1.139866	2.139715	-0.241413
O	0.074594	1.560671	-0.705557
C	0.970906	2.663476	-0.864947
C	0.669781	3.637938	0.303093
N	-2.090951	1.104269	0.159102
C	-1.674702	-0.052258	0.952738
C	-2.603410	-0.329222	2.089527
C	-4.012987	-0.045870	1.912424
N	-4.317871	0.637766	0.726013
C	-3.424306	1.425887	-0.014966
C	-2.134087	-1.008839	3.325760
C	-1.502588	-1.417401	0.064117
N	-0.550610	-1.265726	-1.007356
C	-0.943772	-0.997190	-2.334703
N	-2.298292	-1.070775	-2.659377
C	-3.153956	-1.571976	-1.798998
C	-2.814105	-1.873838	-0.453411
C	0.859612	-1.372467	-0.763270
C	1.392772	-1.060464	0.635724
C	2.804967	-1.613810	0.515760
C	2.600313	-2.864403	-0.371289
O	1.301294	-2.737235	-0.957368
C	2.672581	-4.170092	0.401997
O	1.881619	-4.052370	1.584613
O	3.653251	-0.677821	-0.222783
P	4.104313	0.706016	0.476533
O	5.171963	1.244907	-0.598615
O	-0.118687	-0.733138	-3.185088
N	-4.426290	-1.830988	-2.241376
O	-3.833600	2.327052	-0.717068
O	-4.898580	-0.375875	2.689690
C	2.400245	2.185854	-1.004665
O	2.908978	1.752707	0.281118
O	0.593866	4.942593	-0.265069
O	4.521593	0.582673	1.881497
H	3.367442	-2.871356	-1.154792
H	3.277155	-1.831241	1.471708
H	0.874425	-1.650420	1.393694
H	1.370040	-0.005580	0.889283
H	1.343430	-0.744684	-1.509043
H	-1.122007	-2.157999	0.774129
H	2.448613	1.370641	-1.728858
H	3.021660	3.013085	-1.353004
H	0.719967	3.199475	-1.790992
H	1.456941	3.592182	1.059158
H	-0.517270	2.564098	1.794421
H	-1.386858	3.914076	1.031659
H	-1.627178	2.689769	-1.048628
H	-5.283202	0.925415	0.627432
H	-2.961698	-1.133568	4.022940
H	-1.711713	-1.999552	3.111133
H	-1.335204	-0.434392	3.810393
H	-0.679675	0.162170	1.335960
H	1.890309	-4.899829	2.037671
H	2.299455	-4.969965	-0.248263
H	3.723072	-4.377429	0.650366
H	5.961533	0.695288	-0.659810
H	0.614881	5.585741	0.449924
H	-5.180093	-1.828886	-1.573392
H	-4.638815	-1.459999	-3.155875
H	-3.526430	-2.334819	0.218809

Cartesian Coordinates: *cis-syn* CPD(CpT)

C	-0.481306	3.240280	0.857325
C	-0.918832	2.298945	-0.281547
O	0.283433	1.608324	-0.628193
C	1.232643	2.671347	-0.752006
C	0.966288	3.609988	0.448365
N	-2.024786	1.385979	-0.021223
C	-1.906260	0.347570	0.998503
C	-3.242017	-0.352784	1.418977
C	-4.532510	0.218841	0.881431
N	-4.386019	1.143247	-0.116437
C	-3.223325	1.780219	-0.587462
C	-2.792096	-1.659488	0.639142
C	-3.340203	-1.830362	-0.760438
N	-2.674435	-1.634459	-1.846831
C	-1.288502	-1.412434	-1.843535
N	-0.630830	-1.355461	-0.602183
C	-1.360479	-1.098634	0.596414
C	0.811020	-1.417520	-0.625461
C	1.537161	-1.119516	0.685222
C	2.922719	-1.680082	0.392128
C	2.618054	-2.868454	-0.553851
O	1.237772	-2.765344	-0.905048
C	2.866948	-4.225322	0.080793
O	2.303756	-4.229287	1.391356
O	3.747984	-0.722823	-0.343944
P	4.314731	0.572930	0.430919
O	5.379910	1.124088	-0.642176
O	-0.670539	-1.332651	-2.880736
N	-4.625574	-2.262331	-0.845795
C	-3.351432	-0.583413	2.927134
O	-3.344540	2.654346	-1.418629
O	-5.628498	-0.166766	1.245229
C	2.644122	2.162150	-0.923872
O	3.173473	1.691405	0.338226
O	1.125490	4.943537	-0.024586
O	4.774392	0.333820	1.807181
H	3.246534	-2.770976	-1.447950
H	3.461576	-1.974859	1.289864
H	1.122499	-1.731623	1.488194
H	1.533004	-0.071416	0.971469
H	1.156418	-0.766593	-1.430417
H	-0.801144	-1.468393	1.451338
H	2.661114	1.361039	-1.665100
H	3.275595	2.983609	-1.267552
H	0.998266	3.253337	-1.656200
H	1.670436	3.401132	1.256886
H	-0.497327	2.729798	1.822683
H	-1.114637	4.126799	0.914313
H	-1.241620	2.894451	-1.136047
H	-5.233350	1.489305	-0.549786
H	-4.239809	-1.175086	3.149317
H	-2.472169	-1.107794	3.308030
H	-3.436150	0.369102	3.457180
H	-1.363981	0.766530	1.848264
H	2.381569	-5.116880	1.750957
H	2.403366	-4.987415	-0.556442
H	3.950741	-4.406525	0.116870
H	6.154036	0.556955	-0.732904
H	1.243502	5.523456	0.733323
H	-5.018907	-2.297585	-1.775168
H	-5.260345	-2.126929	-0.073392
H	-2.941683	-2.583386	1.202749

Cartesian Coordinates: *trans-syn* CPD(CpT)

O	-0.069737	-2.198536	-0.260617
C	1.284645	-2.640812	-0.303282
C	1.572560	-3.253539	1.098242
C	0.298310	-3.004424	1.905358
C	-0.754618	-2.901265	0.776134
N	2.145098	-1.533796	-0.709494
C	2.160416	-0.259878	-0.002244
C	3.559552	0.371747	0.229546
C	4.501971	0.109541	-0.940994
N	4.062366	-0.819870	-1.866070
C	3.042326	-1.785663	-1.722716
C	2.917866	1.790374	-0.033584
C	2.487183	2.547193	1.194933
N	1.270436	2.663592	1.608939
C	0.182027	2.247633	0.835490
N	0.430222	1.692652	-0.420940
C	1.708581	1.091204	-0.700215
C	-0.661570	1.443588	-1.374099
O	-1.527088	2.558824	-1.512726
C	-2.922776	2.159361	-1.463735
C	-2.862950	0.629319	-1.679158
C	-1.547810	0.259949	-1.024097
O	-4.016257	-0.108745	-1.193482
P	-4.149389	-1.003404	0.136406
O	-4.247747	-0.342440	1.445776
C	-3.602519	2.602177	-0.156509
O	-3.245859	3.928980	0.171565
O	-0.952978	2.426294	1.241542
N	3.475983	3.142230	1.899766
C	4.273418	0.066453	1.543487
O	3.029529	-2.752693	-2.454891
O	5.555541	0.692670	-1.075419
O	0.487405	-1.774065	2.605077
C	-2.024056	-2.181646	1.183960
O	-2.921293	-2.068930	0.058969
O	-5.444873	-1.857983	-0.262277
H	-0.152330	1.282135	-2.333905
H	-2.843223	0.406085	-2.747711
H	-3.398592	2.671050	-2.303396
H	1.404783	-3.390502	-1.084629
H	0.070869	-3.816465	2.603386
H	-1.005273	-3.915534	0.431304
H	-1.138661	-0.690371	-1.342343
H	-1.666820	0.258376	0.058195
H	-4.684608	2.578636	-0.311759
H	-3.368246	1.921863	0.663533
H	3.520948	2.440811	-0.669869
H	1.838758	1.018938	-1.781632
H	4.436699	3.097164	1.607613
H	3.220985	3.706910	2.695200
H	1.764064	-4.322869	0.995822
H	2.430737	-2.800019	1.592414
H	-2.515600	-2.755788	1.976202
H	-1.791175	-1.190486	1.571316
H	-0.248071	-1.618601	3.206947
H	-5.525513	-2.003272	-1.211920
H	1.625611	-0.386799	0.939536
H	4.704565	-1.026974	-2.622043
H	3.649058	0.327847	2.398614
H	4.502864	-1.001699	1.603811
H	5.217990	0.609372	1.608161
H	-2.346203	3.862710	0.518418

Cartesian Coordinates: *cis-syn* CPD(UpT)

C	0.377049	3.020184	-1.119332
C	0.913976	2.255578	0.100361
O	-0.262793	1.622784	0.625709
C	-1.210485	2.693388	0.684023
C	-0.984001	3.539495	-0.597571
N	2.012787	1.324154	-0.089588
C	1.906383	0.212813	-1.030794
C	3.256797	-0.505878	-1.386241
C	4.533237	0.200610	-0.979216
N	4.370170	1.085784	0.069707
C	3.204357	1.712246	0.519217
C	2.847086	-1.701271	-0.432061
C	3.463411	-1.682944	0.954271
N	2.594319	-1.339396	1.972962
C	1.184403	-1.255993	1.957927
N	0.607542	-1.312462	0.707036
C	1.396131	-1.191286	-0.487120
C	-0.835918	-1.404030	0.666789
C	-1.512487	-1.071681	-0.660771
C	-2.896220	-1.662682	-0.427994
C	-2.591533	-2.910623	0.432393
O	-1.246114	-2.762302	0.901993
C	-2.707680	-4.215066	-0.337573
O	-2.032275	-4.072530	-1.586040
O	-3.723813	-0.759996	0.369281
P	-4.334701	0.555646	-0.339712
O	-5.332294	1.081324	0.806439
O	0.574122	-1.173362	2.998285
O	4.625751	-1.946906	1.162506
C	3.336930	-0.940037	-2.848941
O	3.288791	2.582598	1.359592
O	5.612750	-0.007853	-1.479396
C	-2.615769	2.189036	0.927796
O	-3.188482	1.670828	-0.297650
O	-0.965790	4.903437	-0.186267
O	-4.875676	0.338837	-1.689856
H	-3.283552	-2.932725	1.282225
H	-3.426567	-1.895531	-1.349142
H	-1.063335	-1.642529	-1.473957
H	-1.515705	-0.012996	-0.899902
H	-1.216025	-0.773797	1.471380
H	2.983885	-1.344341	2.907936
H	0.875283	-1.692025	-1.297941
H	-2.603473	1.416647	1.699166
H	-3.240591	3.019154	1.262368
H	-0.955996	3.346750	1.530959
H	-1.780491	3.366259	-1.324267
H	0.231507	2.354739	-1.973126
H	1.032561	3.840653	-1.415514
H	1.279794	2.970706	0.837631
H	5.215613	1.477570	0.465684
H	4.235495	-1.537371	-3.003827
H	2.463762	-1.529458	-3.137981
H	3.395680	-0.068284	-3.505461
H	1.355008	0.553136	-1.908210
H	-2.066683	-4.915337	-2.046484
H	-2.259793	-5.009894	0.270348
H	-3.772383	-4.442733	-0.488152
H	-6.121157	0.536006	0.904423
H	-1.061991	5.457187	-0.966883
H	3.038537	-2.689060	-0.854158

Cartesian Coordinates: *trans-syn* CPD(UpT)

C	1.642221	-3.213297	1.069197
C	1.334908	-2.594776	-0.324811
O	-0.030597	-2.181829	-0.269425
C	-0.701442	-2.951335	0.725821
C	0.343036	-3.066535	1.864248
N	2.165131	-1.469140	-0.729029
C	2.204762	-0.222920	0.021471
C	3.606155	0.422506	0.215141
C	4.470607	0.244617	-1.033454
N	4.010928	-0.688151	-1.952766
C	3.020725	-1.675100	-1.791823
C	2.922524	1.832245	0.094872
C	2.578620	2.463540	1.425746
N	1.224792	2.524336	1.707067
C	0.118310	2.274368	0.869762
N	0.411206	1.712230	-0.345543
C	1.722642	1.150419	-0.605963
C	-0.654297	1.459471	-1.339409
C	-1.519298	0.255039	-1.011509
C	-2.831655	0.597307	-1.689179
C	-2.925901	2.127237	-1.484405
O	-1.534077	2.555598	-1.495079
C	-3.643138	2.572234	-0.202118
O	-3.364090	3.929257	0.077407
O	-3.975250	-0.158706	-1.214865
P	-4.091638	-1.041507	0.125929
O	-4.141173	-0.362616	1.429293
O	-0.991673	2.575605	1.264089
O	3.408882	2.884363	2.198303
C	4.422087	0.063267	1.453490
O	2.981839	-2.619974	-2.551354
O	5.482707	0.874757	-1.232111
C	-1.994324	-2.285569	1.151904
O	-2.879277	-2.120977	0.021624
O	0.455632	-1.866306	2.631847
O	-5.412633	-1.873100	-0.228511
H	-0.109603	1.314797	-2.280483
H	-2.790200	0.368994	-2.755812
H	-3.385788	2.626916	-2.339491
H	1.462633	-3.335858	-1.113289
H	0.145062	-3.923632	2.515232
H	-0.915322	-3.957045	0.334160
H	-1.083445	-0.684150	-1.326341
H	-1.659510	0.235780	0.069108
H	-4.720347	2.484423	-0.365116
H	-3.380009	1.934191	0.643372
H	3.491009	2.563862	-0.479659
H	1.870810	1.134021	-1.686352
H	1.911972	-4.263804	0.951138
H	2.457813	-2.710540	1.587461
H	-2.486626	-2.916530	1.899277
H	-1.789486	-1.314996	1.600761
H	-0.254881	-1.827238	3.280237
H	-5.510476	-2.054787	-1.170451
H	1.713197	-0.385769	0.981524
H	4.614784	-0.850760	-2.750018
H	3.834543	0.194367	2.362504
H	4.749109	-0.979721	1.399934
H	5.305402	0.698357	1.519455
H	-2.452449	3.934866	0.395232
H	0.970408	2.977078	2.576955

Illustration of prominent normal modes

Normal modes of CpT:

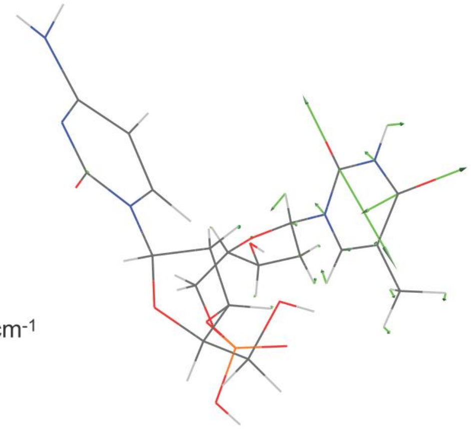

1784 cm^{-1}

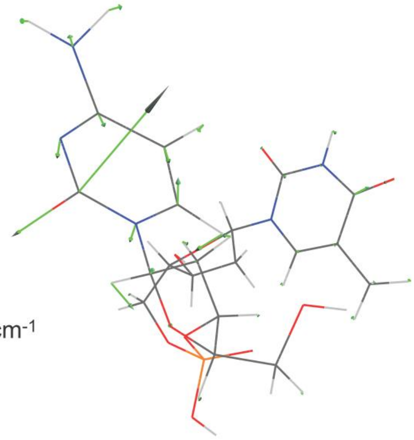

1761 cm^{-1}

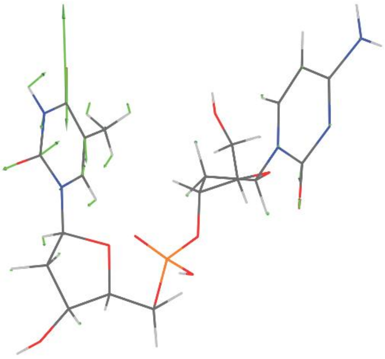

1752 cm^{-1}

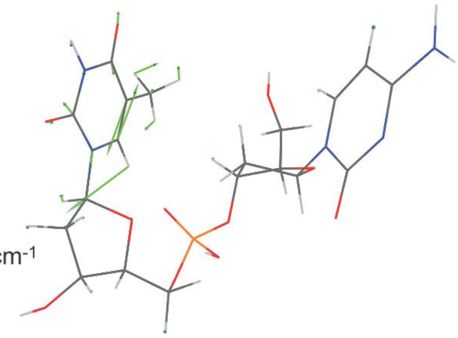

1693 cm^{-1}

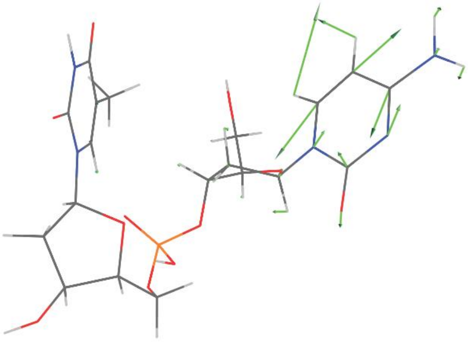

1682 cm^{-1}

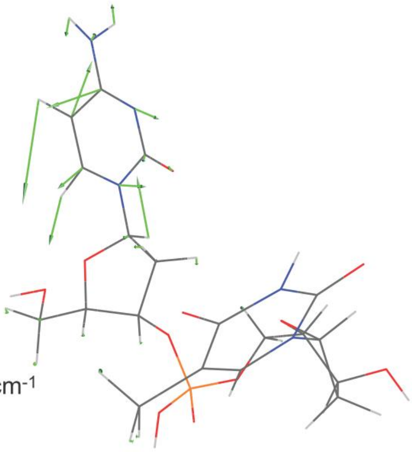

1551 cm^{-1}

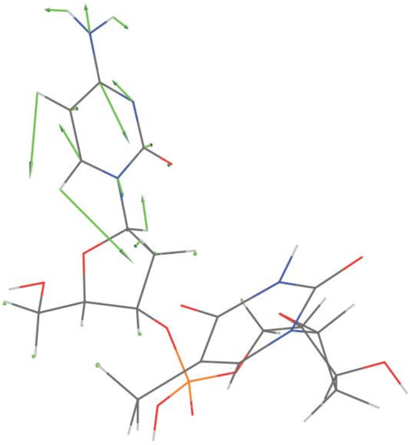

1528 cm^{-1}

Normal modes of Cp^{3}T:

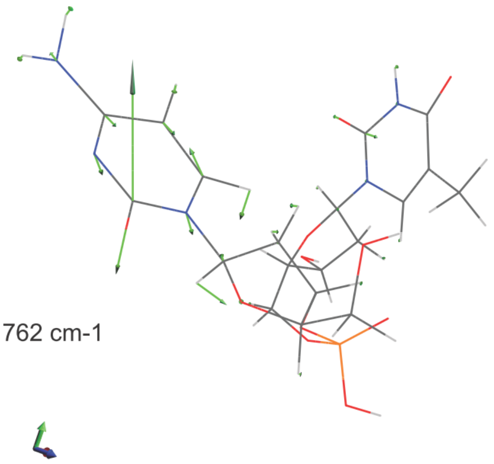

1762 cm-1

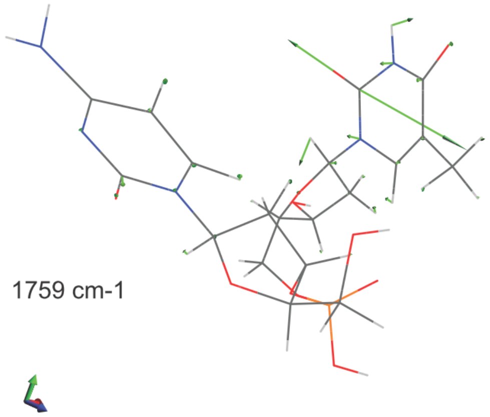

1759 cm-1

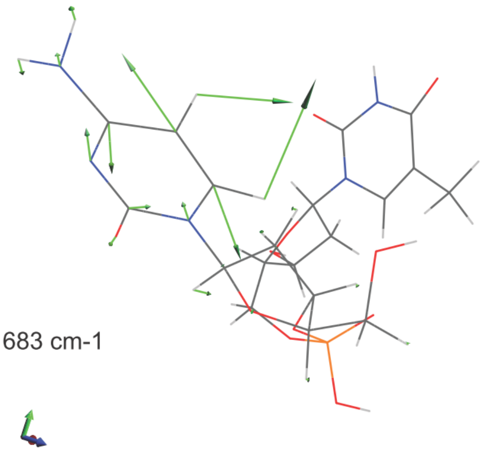

1683 cm-1

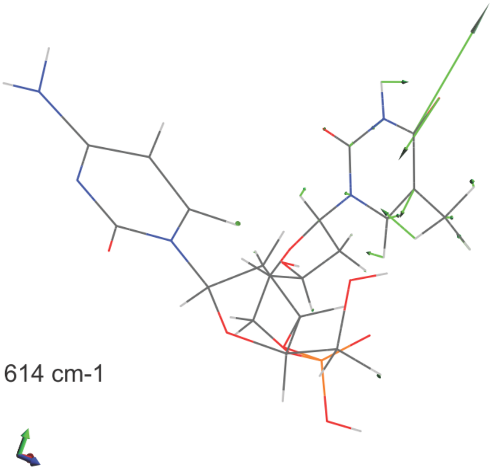

1614 cm-1

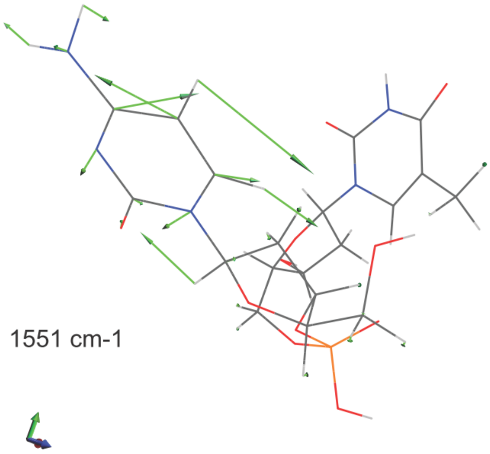

1551 cm-1

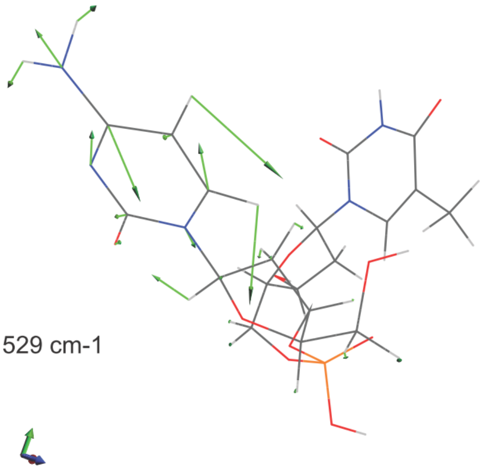

1529 cm-1

Normal modes of BR(CpT):

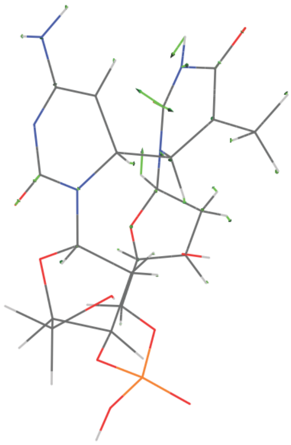
1766 cm-1

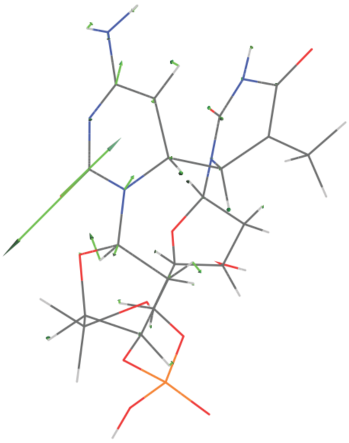
1734 cm-1

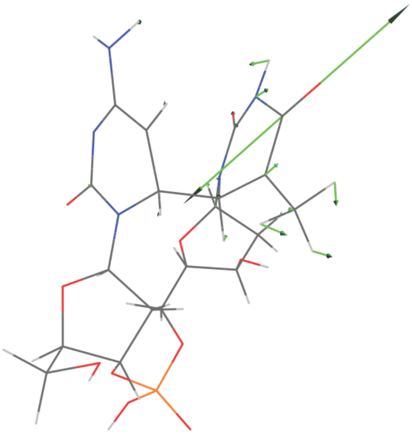
1672 cm-1

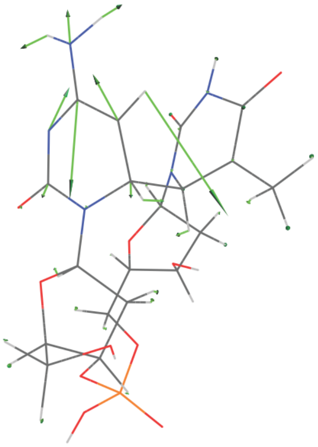
1546 cm-1

Normal modes of *cis*-*syn* CPD(CpT):

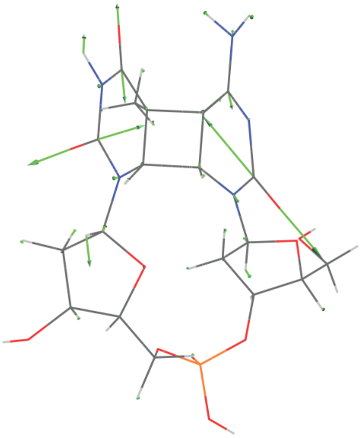

1773 cm-1

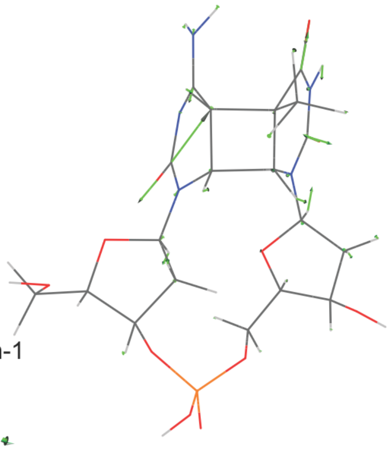

1764 cm-1

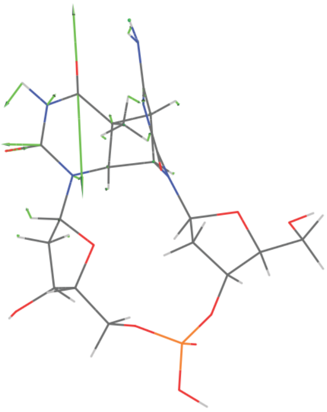

1747 cm-1

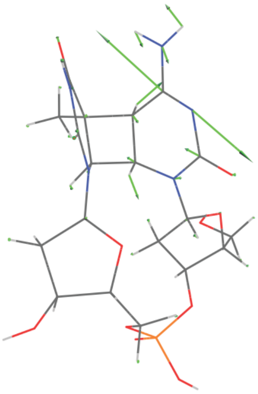

1648 cm-1

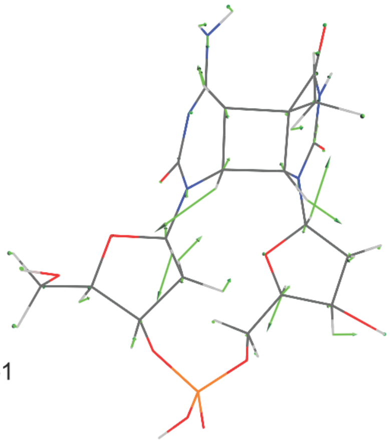

1440 cm-1

Normal modes of *trans*-*syn* CPD(CpT):

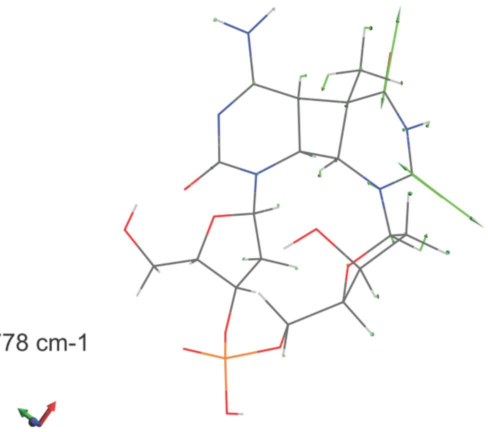

1778 cm-1

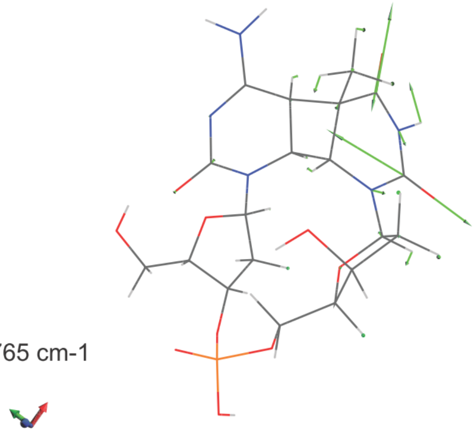

1765 cm-1

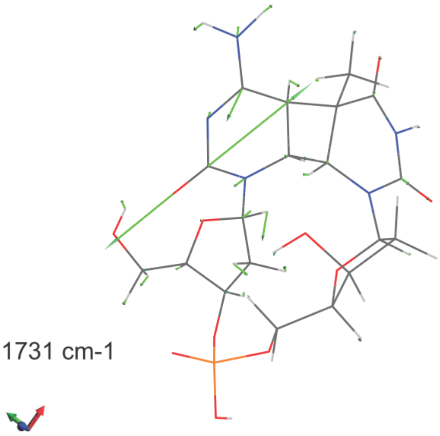

1731 cm-1

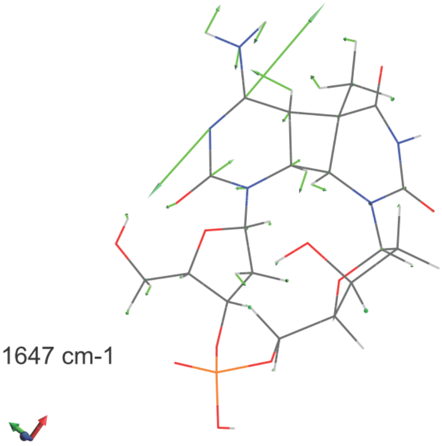

1647 cm-1

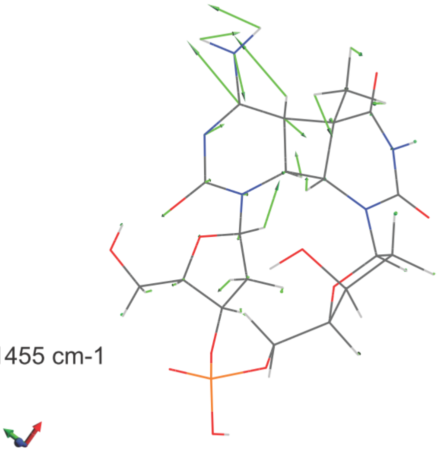

1455 cm-1

Normal modes of *cis-syn* CPD(UpT):

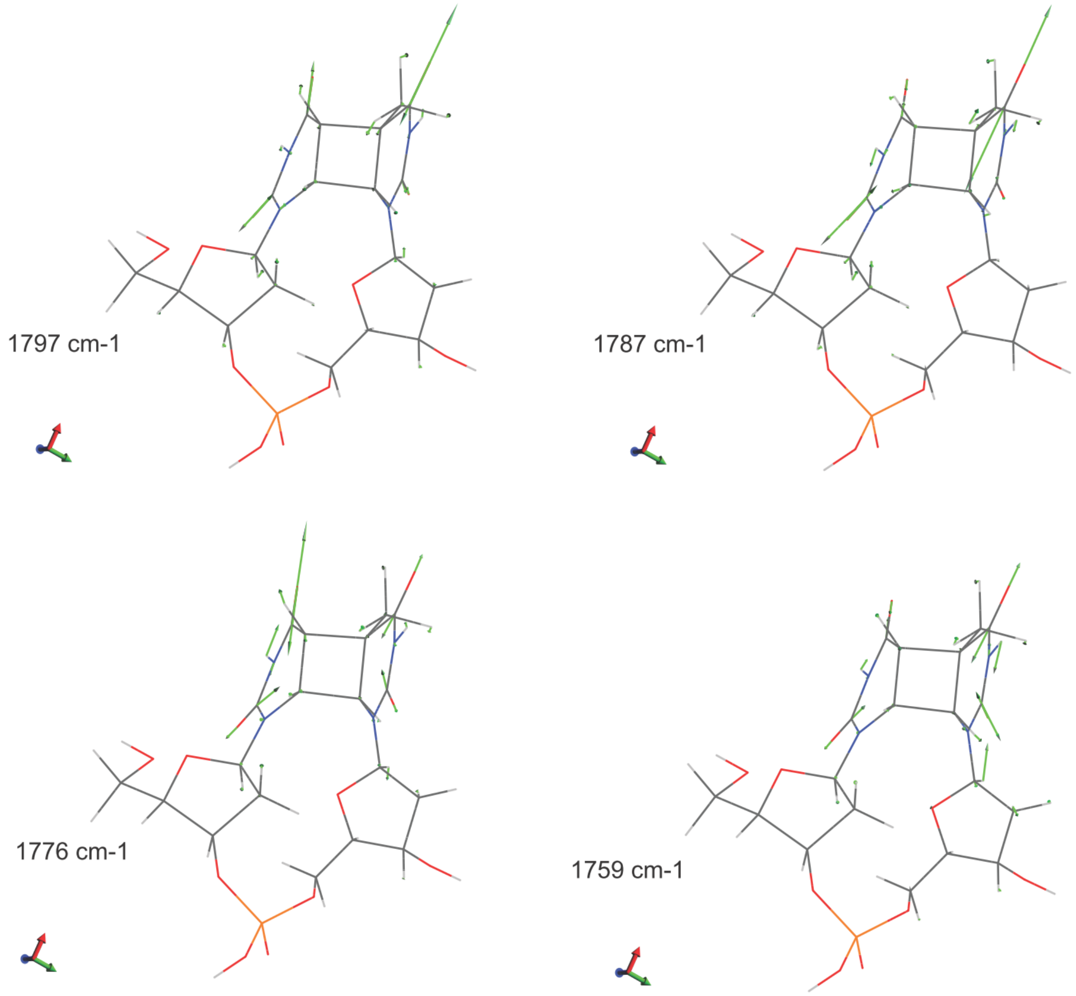

Normal modes of *trans*-*syn* CPD(UpT):

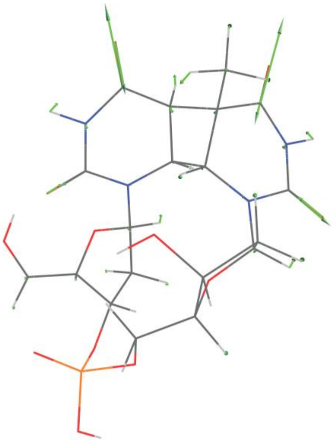

1790 cm-1

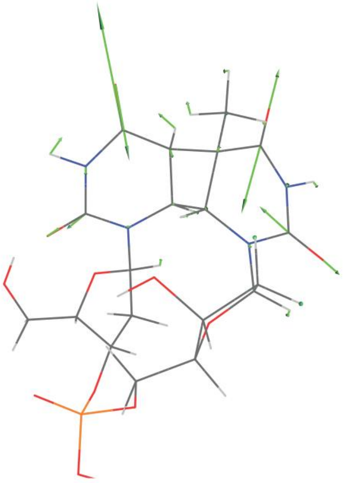

1779 cm-1

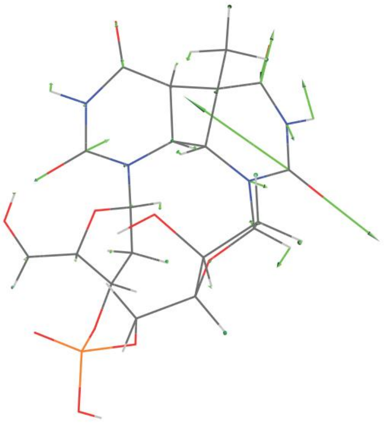

1768 cm-1

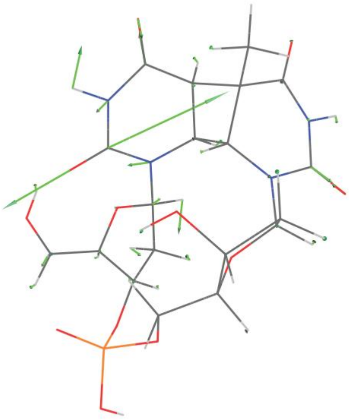

1754 cm-1

3. Time-resolved and stationary spectroscopy of sensitization on CpC

The time-resolved IR measurements were performed on a solution of 2-M (5 mM) and CpC (10 mM) with exclusive excitation of 2-M by nanosecond light pulses at λ = 320 nm. The concentrations were chosen in a range where efficient TTET occurs for a mixture of 2-M and TpT (cf. Ref. [5]). The absorption changes of the mixture (upper panel) are very similar to those of an experiment on the neat sensitizer 2-M published recently by Liu et al. [6]. Also a quantitative analysis of the data by exponential functions does not show any influence of the CpC dinucleosides present in the solution. The absorption changes may be described by a single exponential decay with a time constant of ca. 300 ns. This time constant is in the range found previously for the decay of the triplet state of neat 2-M under ambient conditions. When two exponentials are used in the analysis (time constants of 167 ns and 820 ns) a better fit of the data results.

The decay associated difference spectra (DADS) show only features related to 2-M and the solvent. There is no indication for the involvement of CpC. The present experiment excludes quenching of the triplet state of 2-M by CpC as well as the efficient formation of a cytosine triplet.

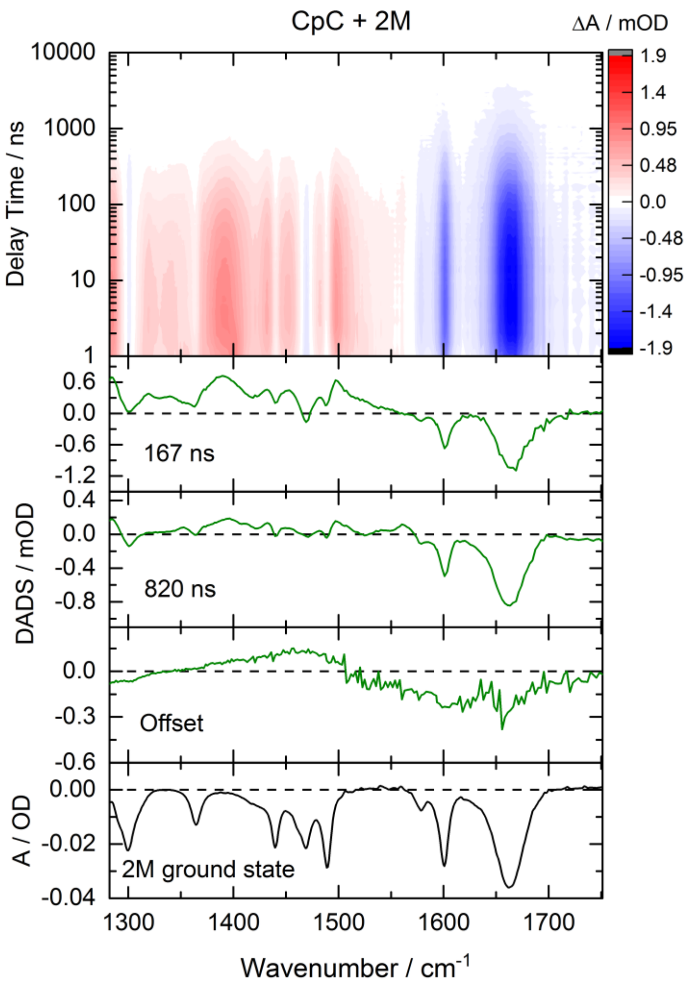

Figure SI3: Time-resolved IR spectra on a solution of 2-M (5 mM) and CpC. Upper panel: Time-resolved IR absorption changes induced by excitation at 320 nm. Central panels: Decay associated difference spectra (DADS) representing a fit using two decay times and an offset. Lower panel: Negative IR spectrum of the sensitizer molecule 2-M.

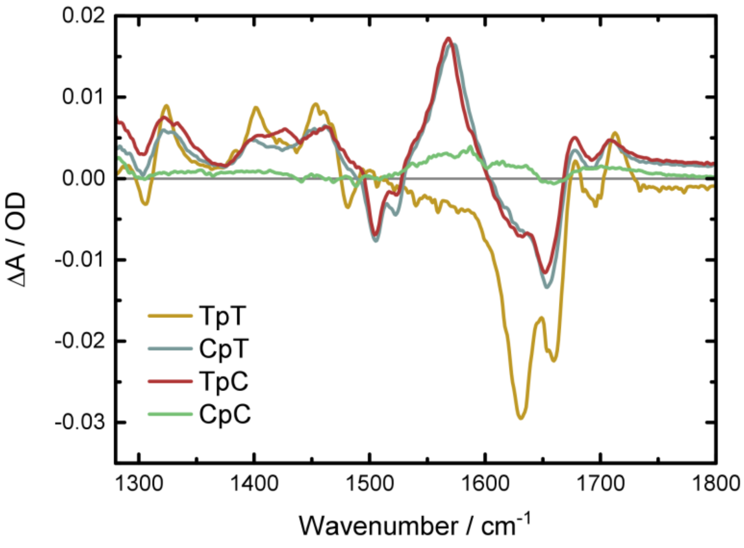

Figure SI4: Absorption difference spectra of mixtures of the dinucleosides TpT, CpT, TpC and CpC with the sensitizer 2-M at an illumination dose of 47 J. For the dinucleosides TpT, CpT and TpC the absorption difference spectra clearly demonstrate the bleach of the initial absoprtion bands due to the formation of the CPD lesions. In addition, one finds induced absorption bands due to CPD formation at low wavenumbers, the pronounced band at 1570 cm^{-1} for CpT and TpC and the characteristic features around 1700 cm^{-1}. For CpC the absorption changes are small. None of the features characteristic for CPD formation are found. One may directly conclude that CPD formation, if it exists, is much less efficient in CpC than in the other dimers.

4. CPD formation yield from the thymine triplet state ^3T

The quantum yield for the formation of the CPD from the thymine triplet state ^3T has been extensively studied for TpT in Ref. [5]. For the mixed dinucleosides CpT and TpC we used a simplified approach to estimate the quantum yields. We measured the illumination induced absorption changes for the mixtures with the triplet donor 2-M as a function of the absorbed illumination dose. The slope of the absorption increase at low doses was determined at the representative band at 1567 cm^{-1}. In order to obtain the extinction coefficient change of this CPD band (1242 $(cm\ M)^{-1}$ for CpT and 1216 $(cm\ M)^{-1}$ for TpC) required for the determination of the reaction yield (see Equation (2) and (3) of Ref. [5]) it was referenced to the absorption bleach of the cytosine band at 1503 cm^{-1}. Other relevant parameters of the experiments are: Sample volume V = 1.3 ml, sample thickness d = 0.1 mm. The TTET efficiency was estimated assuming the same bimolecular collision parameter $\kappa = 1.6 \times 10^9\ (M\ s)^{-1}$ as in the case of TpT or the smaller value of $\kappa = 0.8 \times 10^9\ (M\ s)^{-1}$, considering that only one thymine is present in the mixed dipyrimidines. Taking into account the intersystem crossing efficiency of 2-M of 97% we estimate the CPD formation yield from the thymine triplet ^3T for the two values of κ to be Φ_{3CPD}(CpT) = 1.7% or 1.9% and Φ_{3CPD}(TpC) = 1.2% or 1.3%. Considering the assumptions and approximations mentioned above the accuracy in the determination of the quantum yields should be in the order of 0.5%.

5. UV-Absorption Spectra of CpT, TpC and 2-M

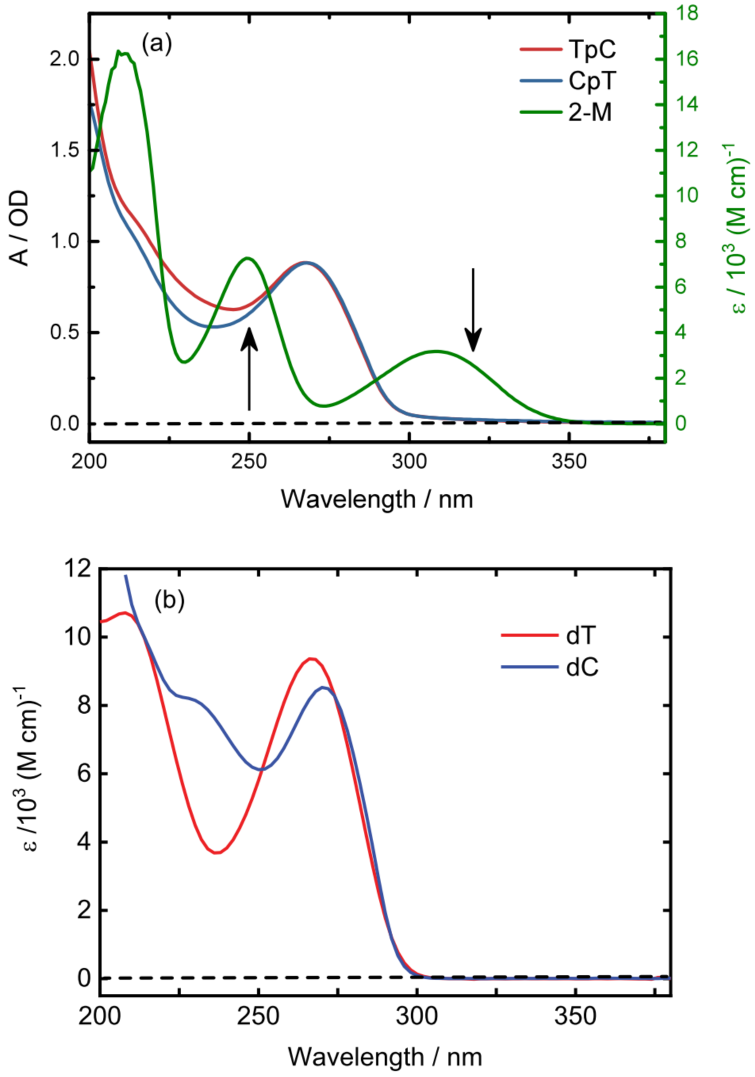

Figure SI5: (a) UV absorption spectra of the dinucleosides TpC and CpT (red and blue, left axis). The extinction coefficient of the photosensitizer 2-M (green) is given in green (right axis). The excitation wavelengths of 250 nm for direct excitation of the dinucleosides and 320 nm for excitation of the photosensitizer are marked by arrows. (b) Extinction coefficient of the nucleosides dT and dC for comparsion. At 250 nm the extinction coefficients are similar, suggesting that at this wavelength thymine and cytosine moieties are excited equally in the dinucleosides.

References

[1] M. J. Frisch, G. W. Trucks, H. B. Schlegel, G. E. Scuseria, M. A. Robb, J. R. Cheeseman, J. Montgomery, J. A., T. Vreven, K. N. Kudin, J. C. Burant, J. M. Millam, S. S. Iyengar, J. Tomasi, V. Barone, B. Mennucci, M. Cossi, G. Scalmani, N. Rega, G. A. Petersson, H. Nakatsuji, M. Hada, M. Ehara, K. Toyota, R. Fukuda, J. Hasegawa, M. Ishida, T. Nakajima, Y. Honda, O. Kitao, H. Nakai, M. Klene, X. Li, J. E. Knox, H. P. Hratchian, J. B. Cross, V. Bakken, C. Adamo, J. Jaramillo, R. Gomperts, R. E. Stratmann, O. Yazyev, A. J. Austin, R. Cammi, C. Pomelli, J. W. Ochterski, P. Y. Ayala, K. Morokuma, G. A. Voth, P. Salvador, J. J. Dannenberg, V. G. Zakrzewski, S. Dapprich, A. D. Daniels, M. C. Strain, O. Farkas, D. K. Malick, A. D. Rabuck, K. Raghavachari, J. B. Foresman, J. V. Ortiz, Q. Cui, A. G. Baboul, S. Clifford, J. Cioslowski, B. B. Stefanov, G. Liu, A. Liashenko, P. Piskorz, I. Komaromi, R. L. Martin, D. J. Fox, T. Keith, M. A. Al-Laham, C. Y. Peng, A. Nanayakkara, M. Challacombe, P. M. W. Gill, B. Johnson, W. Chen, M. W. Wong, C. Gonzalez, J. A. Pople, (Ed.: .), Gaussian, Inc., Wallingford CT, **2004**.

[2] A. D. Becke, *J. Chem. Phys. A* **1993**, *98*, 5648.

[3] V. A. Bloomfield, D. M. Crothers, I. Tinoco, *Nucleic Acids: Structures, Properties, and Functions*, University Science Books, Sausalito, **2000**.

[4] W. Koch, M. C. Holthausen, *A Chemist's Guide to Density Functional Theory*, 2 ed., Wiley-VCH, Weinheim, **2001**.

[5] L. Liu, B. M. Pilles, J. Gontcharov, D. B. Bucher, W. Zinth, *The Journal of Physical Chemistry B* **2016**, *120*, 292.
